# Nutrient regulation of lipochitooligosaccharide recognition in plants via *NSP1* and *NSP2*

**DOI:** 10.1038/s41467-022-33908-3

**Published:** 2022-10-28

**Authors:** Xin-Ran Li, Jongho Sun, Doris Albinsky, Darius Zarrabian, Raphaella Hull, Tak Lee, Edwin Jarratt-Barnham, Chai Hao Chiu, Amy Jacobsen, Eleni Soumpourou, Alessio Albanese, Wouter Kohlen, Leonie H. Luginbuehl, Bruno Guillotin, Tom Lawrensen, Hui Lin, Jeremy Murray, Emma Wallington, Wendy Harwood, Jeongmin Choi, Uta Paszkowski, Giles E. D. Oldroyd

**Affiliations:** 1 Sainsbury Laboratory, University of Cambridge, 47 Bateman Street, Cambridge, CB2 1LR UK; 2 Crop Science Centre, University of Cambridge, 93 Lawrence Weaver Road, Cambridge, CB3 0LE UK; 3 Laboratory for Molecular Biology, Wageningen University & Research, Droevendaalsesteeg 1, 6708 PB Wageningen, the Netherlands; 4 John Innes Centre, Norwich Research Park, Norwich, NR4 7UH UK; 5 Laboratoire de Recherche en Sciences Végétales, Université de Toulouse, CNRS, UPS, Castanet-Tolosan, France; 6 NIAB, 93 Lawrence Weaver Road, Cambridge, CB3 0LE UK; 7 Present Address: NYU-Center of Genomic and System Biology, 12 Waverly Place, New York, NY USA

**Keywords:** Arbuscular mycorrhiza, Strigolactone, Plant signalling

## Abstract

Many plants associate with arbuscular mycorrhizal fungi for nutrient acquisition, while legumes also associate with nitrogen-fixing rhizobial bacteria. Both associations rely on symbiosis signaling and here we show that cereals can perceive lipochitooligosaccharides (LCOs) for activation of symbiosis signaling, surprisingly including Nod factors produced by nitrogen-fixing bacteria. However, legumes show stringent perception of specifically decorated LCOs, that is absent in cereals. LCO perception in plants is activated by nutrient starvation, through transcriptional regulation of *Nodulation Signaling Pathway* (*NSP*)*1* and *NSP2*. These transcription factors induce expression of an LCO receptor and act through the control of strigolactone biosynthesis and the karrikin-like receptor *DWARF14-LIKE*. We conclude that LCO production and perception is coordinately regulated by nutrient starvation to promote engagement with mycorrhizal fungi. Our work has implications for the use of both mycorrhizal and rhizobial associations for sustainable productivity in cereals.

## Abstract (web summary)

Lipochitooligosaccharide (LCO) perception by legumes is required to establish symbiotic relationships with nitrogen fixing bacteria. Here the authors show that nutrient starvation can activate LCO perception in cereals to promote symbiotic association with arbuscular mycorrhizal fungi.

## Introduction

Plant growth and development largely depends on the absorption of mineral nutrients, with nitrogen (N) and phosphorus (P) being the principal limitations to plant productivity^1^. These nutrients are widely applied in agriculture as inorganic fertilizers to maximize crop yields. Fertilizer over-use pollutes terrestrial and aquatic environments, significantly impacts biodiversity, and contributes to a sizable proportion of agricultural greenhouse gas emissions^2–4^.

It is estimated that most land plants associate with beneficial arbuscular mycorrhizal fungi (AMF) that capture mineral nutrients and water from the soil^5–7^. During this association the fungus makes contact with the plant root following recognition of plant-derived strigolactones that promote fungal growth and the production of fungal signals, short-chain chitooligosaccharides (COs), and lipochitooligosaccharides (LCOs)^8–10^. In legumes, the recognition of COs and LCOs activates symbiosis signaling in epidermal cells of the plant root^9–11^, which facilitates the processes necessary to accommodate AMF^12^. In contrast, reports in rice demonstrate recognition of primarily COs^11,13,14^, with very limited perception of LCOs^14^, for establishment of the AMF association. Initial intracellular infection of root atrichoblast cells allows entry to the root, followed by intercellular fungal growth through the root tissue. Within inner root cortical cells, the fungus produces extensively branched arbuscular intrusions, surrounded by plant membrane, creating a large surface interface for nutrient exchange. In parallel extensive fungal colonization of the surrounding soil provides greater access to mineral nutrient capture than the plant root alone can achieve. Sources of N and P captured by the fungus are exchanged for carbon derived from photosynthesis, the sole carbon source that AMF can acquire^15–18^.

Plant species within the N-fixing clade have augmented their symbiotic associations with the addition of interactions with N-fixing bacteria, that allow the plant access to the unlimited supply of atmospheric N through conversion to ammonium. The evolution of this association recruited preexisting processes associated with the AMF association, including symbiosis signaling^19,20^. In AMF-host species, detection of LCOs and/or COs leads to the activation of the common symbiosis signaling pathway, which is essential for accommodating the fungal symbiont^11,21^ and in legumes the same signaling pathway drives the establishment of the interaction with N-fixing bacteria leading to the production of nodules^22–24^. Consequently, it is feasible that manipulation of these pre-existing mechanisms in cereals may enable the engineering of N-fixing symbioses.

Maintaining AMF and rhizobial symbioses are expensive to the plant in terms of supplying sources of carbon from photosynthesis, and as such the symbioses are tightly regulated by the availability of nutrients, particularly N-availability in the regulation of nodulation and P-availability in the regulation of AMF symbioses. Plants preferentially enter these associations when these nutrients are too limiting to be satisfied by direct root capture^25,26^, and it has long been known that in nutrient-replete conditions, the symbioses are strongly inhibited^27,28^. In the case of nodulation, CEP-peptides that act as N-starvation signals, promote the production of miR2111, which inhibits a negative regulator of nodulation in the root^29,30^. P-regulation of the AMF symbiosis is in part controlled by *PHR2*^31,32^, a transcription factor that controls many aspects of the P-starvation response, as well as *NSP1* and *NSP2* that regulate the production of strigolactones^33^. *PHR2* has been demonstrated to directly regulate many genes associated with arbuscule function, in particular nutrient exchange between the plant and the fungus^31^, but to also control the expression of genes associated with CO and LCO perception and the symbiosis signaling pathway^32^, implicating roles both in the early recognition of AMF fungi at the root surface, as well as regulating arbuscule function in the root cortex. Here, we demonstrate the mechanism by which LCO recognition in the plant is coordinately controlled with LCO production by AMF, through the action of *NSP1* and *NSP2*, facilitating nutrient control of AMF colonization.

## Results

### Cereals show non-stringent perception of LCOs

**Fig. 1:**
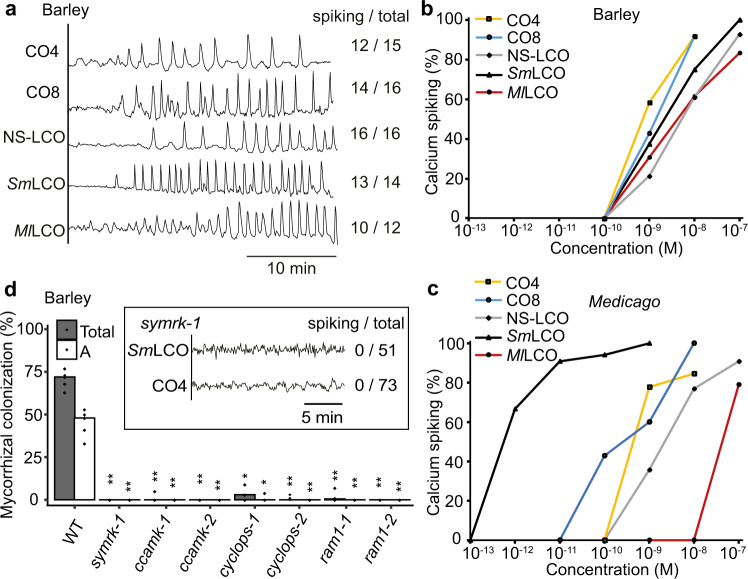
LCOs and COs activate calcium oscillations in barley. **a** Representative calcium traces of atrichoblasts of barley (*H. vulgare*) lateral roots grown in nutrient depletion, responding to 10^−8^ M CO4, 10^−8^ M CO8, 10^−7^ M NS-LCO (non-sulphated LCO produced by *R. irregularis*), 10^−7^ M *Sm*LCO (LCO produced by *S. meliloti*), and 10^−7^ M *Ml*LCO (LCO produced by *Mesorhizobium loti*). Numbers indicate cells responding compared to total cells analyzed. Dose-response curves for LCO and CO induction of calcium oscillations in barley (**b**) and *M. truncatula* (**c**) lateral roots. Calcium spiking (%) indicates the ratio of cells responding with calcium oscillations. **d** AMF colonization measured at 7 weeks post-inoculation of barley wild type (WT), *symrk*, *ccamk*, *cyclops*, and *ram1* mutants, with the inset showing representative traces of barley *symrk* mutant atrichoblasts responding to 10^−7^ M *Sm*LCO and 10^−7^ M CO4. Numbers indicate cells responding compared to total cells analyzed. *n* = 3-5 biologically independent plants. Total: total colonization; A: arbuscules. For statistical analysis a one-sided Wilcoxon test was performed. **p < 0.01.

Whereas legumes show robust induction of symbiosis signaling in response to LCOs and COs^12^, results from rice are inconsistent, with reports of either no LCO responses^11^ or occasional non-periodic calcium oscillations^14^, and inconsistent results on the impact of mutations in the LCO receptor homolog *NFR5*^14,34^. To rationalize the discrepancy of CO and LCO perception in legumes and cereals, we generated an array of cereal lines carrying the calcium reporter cameleon YC3.6, allowing measurement of symbiotic calcium oscillations. At early developmental stages (up to 5-day-old plants) in barley, we only observed CO-induced oscillations, but no responses to LCOs (Supplementary Fig. 1a), consistent with what we previously reported in rice^11^. However, two-week-old barley roots grown under nutrient depletion showed nuclear calcium oscillations in atrichoblasts in response to COs and LCOs (Fig. 1a). Barley perceived LCOs produced by AMF (non-sulfated LCO: NS-LCO), as well as those produced by the N-fixing rhizobial bacteria *Sinorhizobium meliloti* (*Sm*LCO) and *Mezorhizobium loti* (*Ml*LCO). These calcium oscillations are comparable to those induced by LCOs in *Medicago truncatula* and *Lotus japonicus*^12^. Dose response curves reveal that barley shows responses at equivalent concentrations for all molecules tested (Fig. 1b), whereas *M. truncatula* strongly discriminates between LCOs produced by its bacterial symbiont *S. meliloti* versus its non-symbiont *M. loti* (Fig. 1c). Symbiotic calcium oscillations are a function of symbiosis signaling and consistently barley lines mutated in multiple components of this signaling pathway (Supplementary Fig. 1b) abolish associations with the AMF *Rhizophagus irregularis* (Fig. 1d and Supplementary Fig. 1c). *SYMRK*, a co-receptor upstream of calcium oscillations in symbiosis signaling, is essential for both LCO and CO-induced calcium oscillations (Fig. 1d, inset).

**Fig. 2:**
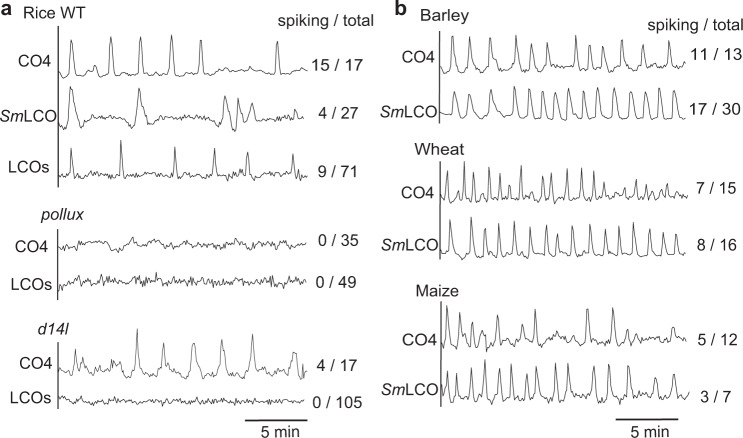
COs and LCOs activate calcium oscillations in cereals. **a** Representative traces of atrichoblasts from lateral roots in rice wild type (WT), *pollux* and *d14l* mutants responding to 10^−^^5^ M CO4, 10^−7^ M *Sm*LCO and LCOs (1:1 mix of 10^−7^ M sulfated-LCO and 10^−7^ M NS-LCO). **b** Representative calcium traces of barley, wheat, and maize atrichoblasts responding to 10^−7^ M CO4 and 10^−7^ M *Sm*LCO. All plants were grown under nutrient deficient conditions. Numbers indicate cells responding compared to total cells analyzed.

We previously reported that rice activates calcium oscillations in response to COs, not LCOs^11^, while others have shown non-periodic responses to LCOs^14^. Our previous work was undertaken in young rice seedlings, the only stage when YC3.6 was detectable in the available rice line and a stage when barley shows only CO responses (Supplementary Fig. 1a). A new rice YC3.6 line allowed analysis at later stages of plants grown under nutrient depletion, and now both LCO and CO responses could be observed, in a manner dependent on *POLLUX*, a component of symbiosis signaling required for calcium oscillations^21,35^ (Fig. 2a). The karrikin receptor *Dwarf14-Like* (*D14L*), has been shown to be essential for the establishment of AMF colonization in rice and acts through the inhibition of the SMAX1 suppressor^36,37^. *d14l* mutants of rice show the complete absence of a transcriptional response to AMF, suggesting a very early role in allowing AMF recognition^36^. Consistent with these earlier observations, we found that LCO responsiveness was abolished in *d14l* and CO responsiveness was attenuated (Fig. 2a).

The observation of LCO induction of symbiosis signaling in both barley and rice suggests that LCO recognition may be a common feature of cereals generally. To assess this, we analysed wheat and maize lines carrying YC3.6 and tested for LCO and CO-induced symbiotic calcium oscillations. We observed responses comparable to that observed in barley in both wheat and maize (Fig. 2b). We conclude that CO and LCO induction of symbiosis signaling are a common feature of legumes and cereals, with legumes augmenting the stringency of LCO recognition to differentiate rhizobial species.

### Nutrient regulation of LCO recognition involves *NSP1* and *NSP2*

**Fig. 3:**
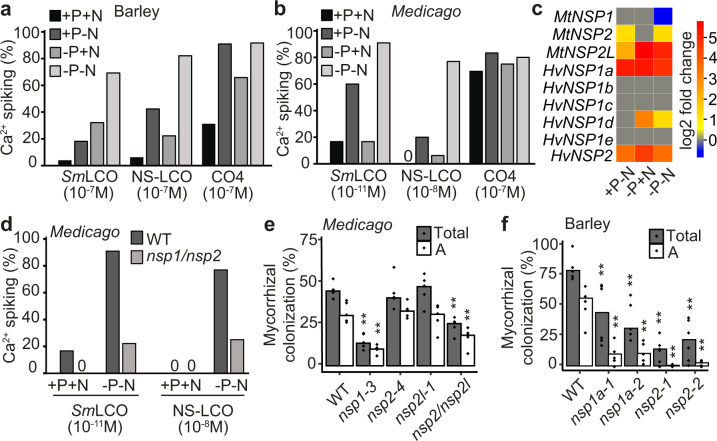
*NSP1* and *NSP2* control nutrient regulation of symbiosis signaling and AMF colonization. Nutrient regulation of symbiosis signaling in 16-day-old barley (**a**) and *M. truncatula* (**b**) lateral roots grown under different nutrient regimes, responding to *Sm*LCO, NS-LCO, or CO4. Calcium spiking (%) indicates the number of cells responding with calcium oscillations, with 0 indicating no calcium oscillations observed. **c** A heatmap showing the expression of *M. truncatula* and barley *NSP1* and *NSP2* homologs upon N- and/or P- starvation. +P-N represents the comparison of gene expression under +P−N to +P + N ( + P−N/ + P + N), similarly −P + N and −P-N representing −P + N/ + P + N and −P−N/ + P + N. **d** The percentage of *M. truncatula* atrichoblasts on lateral roots showing calcium oscillations in wild type and the *nsp1-1/nsp2-2* double mutant, grown under nutrient replete or deplete conditions. **e** AMF colonization of *M. truncatula* mutants grown under P-limited conditions, measured at 3 weeks post inoculation. *n* = 5 biologically independent samples. **f** Root-length colonization of barley wild type*, nsp1a*, and *nsp2* mutants grown under P-limited conditions, measured at 7 weeks post inoculation. *n* = 5–6 biologically independent plants. Total: total colonization; A: arbuscules. *p*-values for colonization levels were determined by a one-sided Wilcoxon test. **p < 0.01; *0.01 < *p* < 0.05.

We only observed LCO recognition in barley when the plants were depleted for nutrients and the onset of LCO responsiveness was associated with developmental markers of nutrient starvation (Supplementary Fig. 1d). We, therefore, further assessed the regulation of symbiosis signaling under different nutrient regimes. Barley and *M. truncatula* only show activation of LCO perception under N- and/or P-starvation (Fig. 3a, b), but the oscillation patterns are not equivalent among each nutrient regime, with the combination of N- and P-starvation displaying more frequent and robust oscillations (Supplementary Fig. 2a, b). The principal difference between the barley and *M. truncatula* responses to nutrient starvation is a strong preference for N-starvation in LCO perception in *M. truncatula* (Fig. 3b), reflecting the additional importance of LCO regulation in the control of the association with N-fixing bacteria. We found no impact of nutrient starvation for CO induction of calcium oscillations in *M. truncatula*, but some nutrient regulation of the CO response in barley (Fig. 3a, b). However, the degree of nutrient regulation of CO-induced calcium oscillations in barley was considerably less than that observed for LCO responses (Fig. 3a).

*Nodulation Signaling Pathway* (*NSP*)*1* and *NSP2* were discovered because of their major roles in establishing root nodule symbiosis in *M. truncatula*^38,39^, but were secondarily shown to also be necessary for appropriate AMF colonization^9,40,41^. Among the *NSP1* and *NSP2* gene families in *M. truncatula* and barley are a number of genes regulated by the nutrient status (Fig. 3c and Supplementary Fig. 3a, b), suggesting a possible role in the nutrient regulation of symbiosis. The promotion of LCO responsiveness by nutrient starvation, measured by activation of calcium oscillations, was greatly attenuated in *M. truncatula nsp1/nsp2*, compared to wild type (Fig. 3d). That this is relevant to symbiosis is supported by the observation that *M. truncatula nsp1* shows defects in AMF colonization (Fig. 3e and Supplementary Fig. 4a–b), as previously reported^41^. However, no colonization defects were observed in single mutants of *M. truncatula NSP2* or its homolog *NSP2L* (Fig. 3e and Supplementary Fig. 4a–c), but a double mutant between these two genes, *nsp2/nsp2l*, displayed reduced AMF colonization (Fig. 3e). Barley possesses only a single ortholog of *NSP2* (Supplementary Fig. 3b), that is transcriptionally responsive to nutrient starvation (Fig. 3c), but five potential orthologs of *NSP1* (Supplementary Fig. 3a), one of which *NSP1a* shows a very strong transcriptional response to nutrient starvation (Fig. 3c). Mutation of *HvNSP1a* and *HvNSP2* revealed major functions in AMF colonization, with reduced total colonization and arbuscule formation (Fig. 3f and Supplementary Fig. 5a). The *nsp* mutant phenotypes in barley are more severe than any of the *M. truncatula nsp* mutants (Fig. 3e, f), revealing a major function for these genes during AMF associations in cereals.

**Fig. 4:**
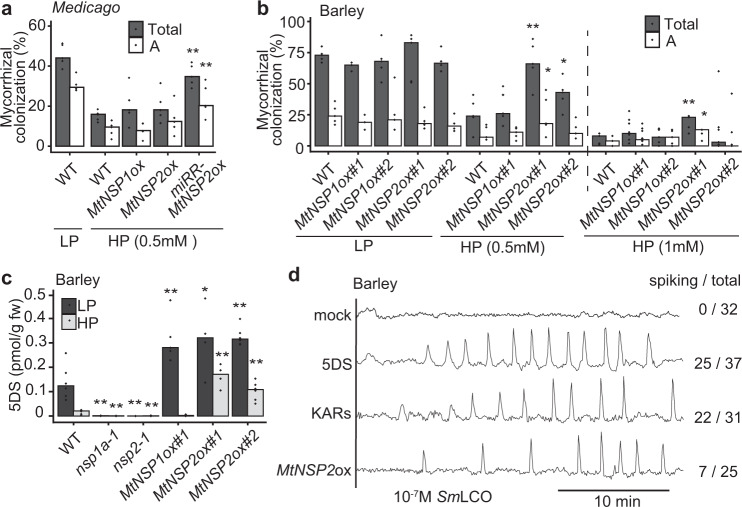
Overexpression of *NSP2* promotes AMF colonization and strigolactone biosynthesis. **a** AMF colonization measured at 5 weeks post-inoculation in *M. truncatula* roots overexpressing *MtNSP1*, *MtNSP2,* and a miR171h-resistant version of *NSP2* (*miRR-MtNSP2*), grown under high P (HP) conditions, with wild-type (WT) under low P (LP) and HP conditions as controls. WT indicates roots transformed with an empty vector. *n* = 5 biologically independent samples. **b** Root-length colonization at 7 weeks post-inoculation in barley roots overexpressing *MtNSP1* and *MtNSP2* grown under different P levels. *n* = 3–5 biologically independent plants. **c** Strigolactone levels in barley *nsp* mutants and *NSP* overexpression lines under both LP and HP conditions. *n* = 4–6 biologically independent samples. **d** Representative calcium traces of atrichoblasts on lateral roots of barley responding to *Sm*LCO. Wild-type plants were grown under repressive P-conditions and pretreated for 16 h with buffer (mock), 0.1 μM 5-deoxystrigol (5DS), or a mixture of 0.1 μM karrikin 1 and karrikin 2 (KARs), as well as transgenic plants overexpressing *MtNSP2* grown under repressive P-conditions. Numbers indicate cells responding with calcium oscillations compared to total cells analyzed. Total: total colonization; A: arbuscules. *p*-values were determined by a one-sided Wilcoxon test. **p < 0.01; * 0.01 < *p* < 0.05.

*NSP2* in *M. truncatula* is controlled by miR171h and, consistent with previous work that overexpression of a miR171h-resistant version of *NSP2* (*miRR-NSP2*), but not native *NSP2*, increased AMF colonization under P-limiting condition^40^, we observed that only *miRR-NSP2* overexpression promoted AMF colonization in *M. truncatula* under repressive P concentrations (Fig. 4a), while native *NSP1* or *NSP2* overexpression showed no effect (Fig. 4a). In wild-type barley, AMF colonization is suppressed with increased P concentrations (Supplementary Fig. 6a), and overexpression of *M. truncatula* (*Mt*)*NSP2*, but not *MtNSP1* (Supplementary Fig. 6b–c), in barley also partially blocked P-suppression of mycorrhization (Fig. 4b and Supplementary Fig. 6d). We conclude that the mis-regulation of *NSP2* is sufficient to partially overcome P-suppression of mycorrhization in both *M. truncatula* and barley, pointing towards a conserved dominance of *NSP2* in the control of the AMF symbiosis.

### *NSP1* and *NSP2* control strigolactone biosynthesis in response to nutrient starvation

P-starvation is associated with the biosynthesis of strigolactones^42–44^, in part controlled by *NSP1* and *NSP2*^33,45^ and this is necessary for mycorrhizal and rhizobial colonization^46,47^. Consistently, we found barley *nsp1* and *nsp2* mutants lost all P starvation-induced strigolactone production (Fig. 4c). Overexpression of both *MtNSP1* and *MtNSP2* in barley led to higher levels of strigolactone at low P, but only *MtNSP2* overexpression led to higher levels of strigolactone at high P (Fig. 4c). To assess whether such strigolactone induction could explain the promotion of LCO recognition at repressive P, we grew barley, *M. truncatula* and wheat at high P and treated roots overnight with either 5-deoxystrigol (5DS) or a combination of karrikin 1 and karrikin 2 (KARs). Either treatment was sufficient to strongly promote LCO recognition at a suppressive P concentration (Fig. 4d and Supplementary Fig. 2c, d), with a combination of karrikins showing a greater effect than single karrikin treatment (Supplementary Fig. 2e). A minimum of 8 h of treatment was required to see an effect (Supplementary Table 1). Moreover, overexpression of *MtNSP2* in barley also allows LCO perception under repressive P conditions (Fig. 4d). We conclude that *NSP1* and *NSP2* are necessary and sufficient for P-regulation of strigolactone biosynthesis and the promotion of LCO recognition.

**Fig. 5:**
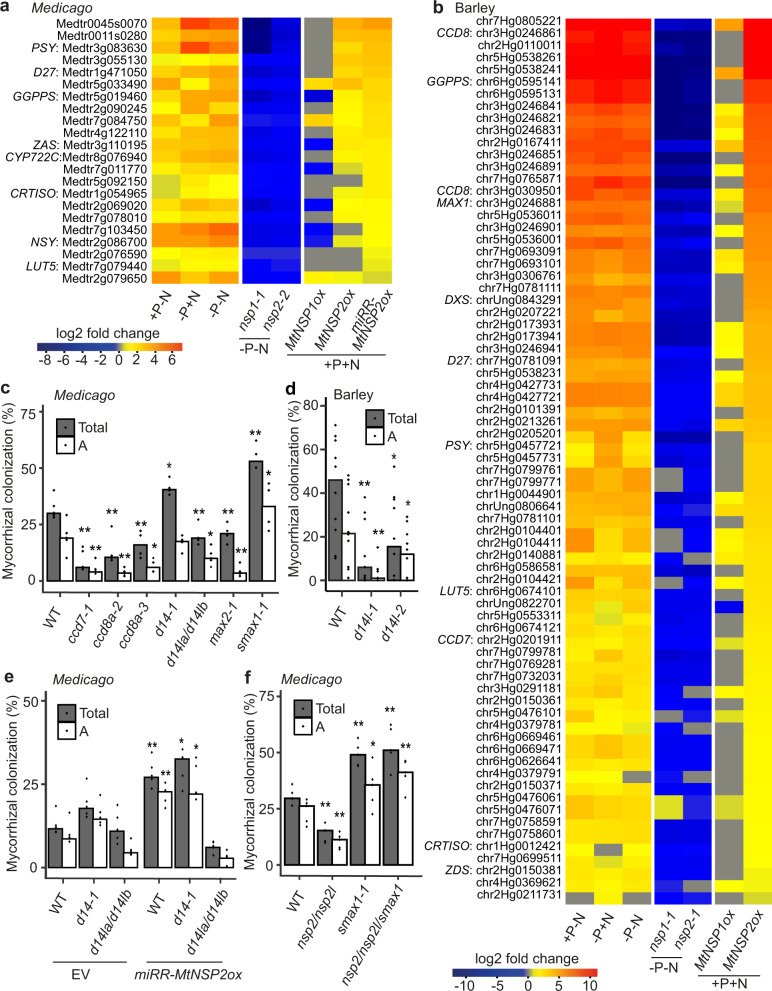
NSP2 overrides P-suppression via strigolactone production and D14L/SMAX1 signaling. Heatmaps showing N- and/or P-starvation induced genes regulated by *NSP1* and *NSP2* and constitutively activated by miRNA-resistant *MtNSP2* overexpression in *M. truncatula* (**a**) and *MtNSP2* overexpression in barley (**b**). Genes involved in strigolactone biosynthesis are annotated. +P–N, −P + N, and −P−N represent the expression of these starvation-induced genes in wild-type plants by comparing −N or/and −P conditions to +P + N. The *nsp* mutants show gene expression in *nsp* mutants compared to wild-type plants under nutrient depletion, while *NSPox* shows *NSP* overexpression roots compared to wild type under nutrient-replete condition. **c** AMF colonization at 3 weeks post-inoculation of *M. truncatula* mutants defective in strigolactone biosynthesis and signaling, as well as karrikin signaling. Plants were grown under low P conditions. *n* = 4–5 biologically independent samples. **d** Root-length colonization at 7 weeks post-inoculation in barley wild type and *d14l* mutant roots under P deficient conditions. *n* = 10 biologically independent plants. **e** AMF colonization of wild type, *d14*, and the *d14l* double mutant with or without overexpression of *miRR-MtNSP2*. Plants were grown under high-P (0.5 mM PO_4_^3−^) conditions for 5 weeks. EV: empty vector. *n* = 5 biologically independent samples. **f** AMF colonization at 3 weeks post inoculation of *M. truncatula nsp2/nsp2l*, *smax1*, and *nsp2/nsp2l/smax1* triple mutants. Plants were grown under low-P conditions. *n* = 5 biologically independent samples. Total: total colonization; A: arbuscules. For statistical analysis, a one-sided Wilcoxon test was performed. **p < 0.01; *0.01 < *p* < 0.05.

To assess the contributions made by *NSP1* and *NSP2* to nutrient starvation, we analyzed the N- and P-starvation transcriptome in barley and *M. truncatula nsp* mutants. *NSP1* is responsible for 12% of the N- and P-starvation transcriptome in *M. truncatula* and 15% in barley, while *NSP2* is responsible for 18% in *M. truncatula* and 23% in barley (Supplementary Fig. 7 and Supplementary Table 2). In order to focus on those genes most directly regulated by these transcription factors, we selected the nutrient responsive genes across all starvation conditions that were *nsp1* or *nsp2-*dependent, as well as induced by *NSP2* overexpression (Fig. 5a, b). This focused group of *NSP*-regulated genes includes many enzymes involved in apocarotenoid biosynthesis (Supplementary Fig. 8a), including most of the strigolactone biosynthesis pathway, but also many enzymes involved in other apocarotenoids associated with mycorrhization: mycorradicins, blumenols, and zaxinones^48^. In *M. truncatula*, *nsp1* mutants loose root expression of the strigolactone biosynthetic gene *D27* (Supplementary Fig. 8b), as previously reported^33,45^, but overexpression of *NSP1* alone is not sufficient to activate *D27* or other strigolactone biosynthetic genes (Fig. 5a and Supplementary Fig. 8c). Rather overexpression of *NSP2* alone has some gene induction ability, but a combination of *NSP1* and *NSP2* overexpression in *M. truncatula* maximally activates these genes (Supplementary Fig. 8c), revealing the combinatorial function of NSP1 and NSP2 in *M. truncatula*^49^. Of the focused group of *NSP*-regulated genes, 73% and 44% could function in apocarotenoid or small molecule biosynthesis in *M. truncatula* and barley, respectively (Fig. 5a, b). The apocarotenoid and associated pathways appear a principal target of *NSP* action, and thus we propose that component(s) of this pathway may explain *NSP* action in nutrient regulation of symbiotic perception.

### *NSP2* promotes AMF colonization through karrikin signaling

Both karrikin and strigolactone treatment overcame P-suppression of LCO recognition (Fig. 4d and Supplementary Fig. 2c, d), and these two molecules are perceived by different, but homologous receptors: D14L and D14, respectively. Mutations in the strigolactone biosynthesis genes *CCD7* and *CCD8a* (two *CCD8* homologs in *M. truncatula*, Supplementary Figs. 3c, 7b and 4b) show strong reductions in *R. irregularis* colonization (Fig. 5c), while mutants in *D14* (Supplementary Figs. 3d, 4a and 4b) show enhanced *R. irregularis* colonization, although the significance of this effect was variable between experiments (Fig. 5c and Supplementary Fig. 4d). This suggests strigolactone biosynthesis is important for the AMF symbiosis, but perception by D14 is not required and may even have negative connotations for AMF colonization. In contrast, mutants in the karrikin receptor *D14L* (two copies in *M. truncatula* and a single ortholog in barley, Supplementary Fig. 3d) display decreased colonization by *R. irregularis* in both *M. truncatula* and barley (Fig. 5c, d and Supplementary Fig. 5b)*. MAX2/D3*, encoding an F-box protein crucial for both strigolatone and karrikin-like signaling, is a positive regulator of AMF colonization, with its *M. truncatula* mutant (Supplementary Fig. 4a, b) showing reduced *R. irregularis* colonization (Fig. 5c). Consistently, mutants in the negative regulator *SMAX1* (Supplementary Figs. 3e, 4a and 4b), that is targeted for degradation by D14L^37^, show enhanced *R. irregularis* colonization (Fig. 5c). While not equivalent in genetic penetrance, the mycorrhizal phenotypes of these biosynthesis and signaling genes in *Medicago* are comparable to what has been observed in rice and other species^36,37,47,50,51^. We conclude that strigolactones are required as secreted signals to AMF in the rhizosphere, but plant recognition of strigolactones through D14 has no role in this symbiosis. In contrast, the D14L receptor, that is essential to remove the SMAX1 suppressor, shows comparable positive regulation of AMF establishment, with SMAX1 negatively regulating this association.

*D14L* is required in rice to promote LCO recognition for activation of symbiotic calcium oscillations (Fig. 2a), suggesting a role for this signaling component in the establishment of AMF perception at the root surface. Considering that *NSPs* regulate enzymes involved in apocarotenoid biosynthesis (Fig. 5a, b) and also control nutrient-starvation induced promotion of LCO perception (Fig. 3d), we hypothesized that *NSP1* and *NSP2* may function in nutrient starvation through the production of the D14L ligand. To test this, we assessed *NSP2* promotion of AMF colonization in *d14* and *d14l* mutants. We found that the promotion of AMF colonization at restrictive P-concentrations by *NSP2* overexpression was dependent on *D14L*, but not *D14* (Fig. 5e). Consistently, AMF colonization of an *nsp2/nsp2l/smax1* triple mutant resembled the higher colonization of *smax1*, rather than the low colonization observed in the *nsp2/nsp2l* double mutant (Fig. 5f). From this we conclude that *NSP2* functions upstream of the *D14L/SMAX1* signaling module.

Strigolactones have been reported to have negative and positive effects in the regulation of nodulation in pea, dependent on whether mutants in biosynthetic enzymes or the receptor were analyzed^47,52^. In *M. truncatula* we found that both *ccd7* and *ccd8a* mutants develop more nodules (Supplementary Fig. 4e), reflecting a negative role for strigolactones in nodulation of *M. truncatula*. Consistently, strigolactone signaling, through *D14*, also represses nodulation, while karrikin-like signaling, through *D14L* has no impact (Supplementary Fig. 4e). We conclude that the positive effects of strigolactone biosynthesis and karrikin-like signaling are specific to the AMF symbiosis and do not have equivalent functions during the association with N-fixing bacteria.

### *NSP1* and *NSP2* control nutrient starvation-induction of an LCO receptor in barley

**Fig. 6:**
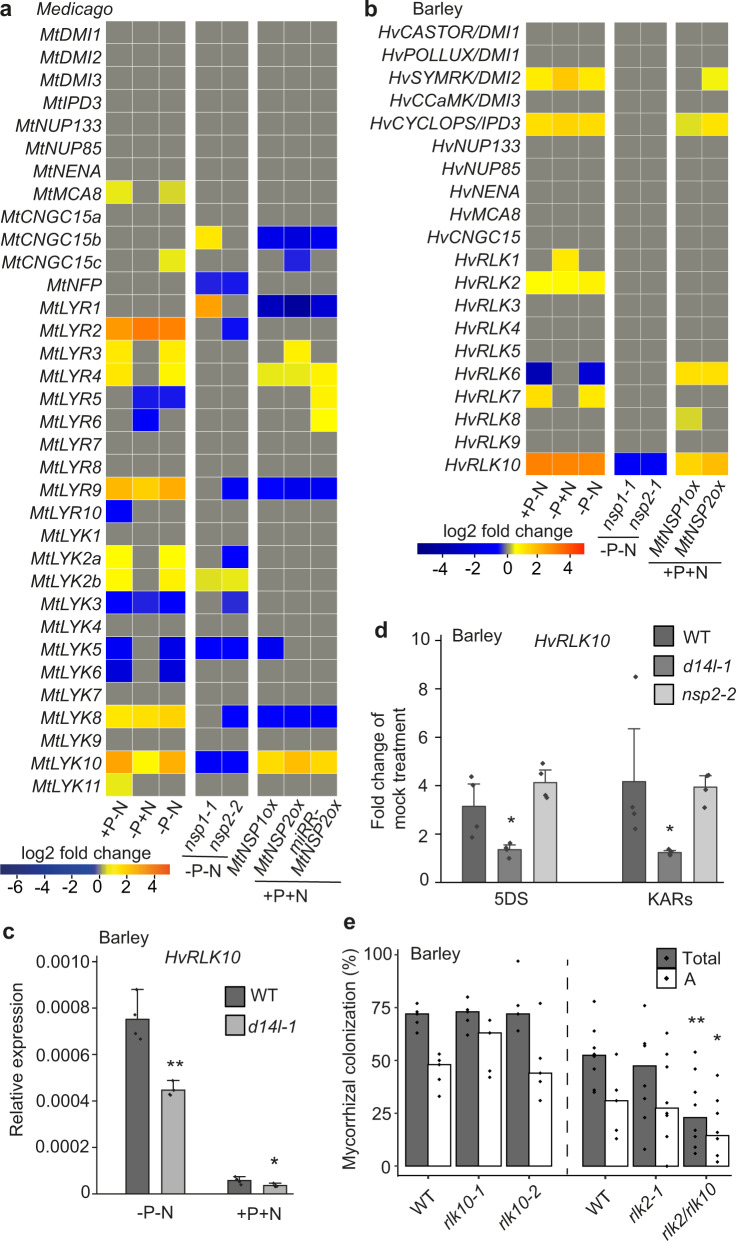
*NSP1* and *NSP2* promote the expression of LysM-type receptors allowing LCO recognition. Heatmaps representing the expression of genes encoding the common symbiosis signaling pathway and LysM-type receptor-like kinases in wild-type plants under N- and/or P-starvation, as well as in *nsp* mutants under nutrient deficient conditions and in *NSP* overexpression lines under nutrient-replete conditions in *M. truncatula* (**a**) and barley (**b**). +P−N, −P + N and -P-N represent the expression of these genes in wild-type plants under −N or/and −P conditions in comparison to +P + N conditions. *nsp* mutant columns show the expression of these genes in *nsp* mutants compared to wild type under nutrient-deficient conditions, while *NSPox* columns show *NSP* overexpression roots compared to wild type under nutrient-replete conditions. **c** Expression of barley *RLK10* in wild type and *d14l* mutant roots. Plants were grown in nutrient deficient or sufficient conditions for 3 weeks. *n* = 4 biologically independent samples, ±s.e.m.; **indicates *p* < 0.01, *indicates 0.01 < *p* < 0.05, measured using a Student’s *t*-test (one-tailed, two-sample equal variance). **d** Induction of barley *RLK10* by 5DS and KARs. Wild type, *d14l-1* and *nsp2-2* mutant plants were grown under repressive P-conditions and pretreated for 2 days on solid media containing 1 μM 5-deoxystrigol (5DS) or a mixture of 1 μM karrikin 1 and karrikin 2 (KARs). *n* = 4 biologically independent samples, ±s.e.m. *indicates 0.01 < *p* < 0.05, measured using a Student’s *t*-test (one-tailed, two-sample equal variance). **e** Root-length colonization of barley *rlk10*, *rlk2*, and *rlk2/rlk10* double mutants grown under P-limited conditions, measured at 7 weeks post inoculation. The *RLK10* mutation in the *rlk2/rlk10* double mutant is equivalent to *rlk10-1*. *n* = 5–8 biologically independent plants. Total: total colonization; A: arbuscules. *p*-values for colonization levels were determined by a one-sided Wilcoxon test. **p < 0.01; *0.01 < *p* < 0.05.

Previous work has reported that mutation of *SMAX1* upregulates genes involved in the symbiosis signaling pathway, including the candidate LCO receptor *NFR5*, as well as *SYMRK*, *CCaMK*, and *CYCLOPS*^37^. Our finding that *NSP2* functions through activation of *D14L* signaling and thus likely removal of SMAX1, implicates a potential role of NSP2 in regulating symbiosis signaling. Overexpression of *MtNSP2* in barley significantly promotes the expression of *SYMRK* and *CYCLOPS*, as well as *RLK10* (the *NFR5* homolog in barley, Supplementary Fig. 3f), under nutrient replete conditions (Fig. 6b). Notably, while these genes are regulated by nutrient starvation, only *RLK10* shows nutrient-starvation activation in a manner dependent on *NSP1* and *NSP2* (Fig. 6b), as well as *D14L* (Fig. 6c). 5DS and KARs treatments allow LCO perception under repressive P conditions (Fig. 4d) and consistently also promote expression of *RLK10* (Fig. 6d and Supplementary Fig. 9b), in a manner dependent on *D14L*, but independent of *NSP2* (Fig. 6d). These data reveal that the action of 5DS and KARs are both dependent on *D14L*, showing, at least in this experimental set-up, that D14L signaling can be promoted by 5DS, as well as KARs.

Our data suggest that *NSP* and *D14L* control of AMF colonization, as a function of nutrient status, may principally be through the regulation of *RLK10*. To test this we generated mutants in *RLK10* and its close homolog *RLK2* (Supplementary Fig. 3f), which is also upregulated under nutrient starvation (Fig. 6b and Supplementary Fig. 9a). No significant defect of AMF colonization was observed in the *rlk10* or *rlk2* single mutants (Fig. 6e and Supplementary Fig. 9c, d), but a combination *rlk2*/*rlk10* double mutant was significantly impaired in AMF colonization (Fig. 6e and Supplementary Fig. 9d). These two receptors contribute to LCO and CO perception with *rlk2* attenuated in both LCO and CO-induced calcium oscillations and the *rlk2/rlk10* double mutant completely insensitive to LCO perception (Table 1). We conclude that *RLK10* acts as a LCO receptor and its nutrient regulation, in combination with that of *RLK2*, allows LCO recognition under nutrient starvation conditions.

**Table 1:** *RLK2* and *RLK10* contribute to LCO and CO perception for induction of calcium spiking (cells spiking/total cells analysed)

	10^−7^ M *Sm*LCO (spiking/total)	10^-7 ^M CO4 (spiking/total)
WT	54 / 72	52 / 69
*rlk2*	12 / 110	14 / 88
*rlk10*	38 / 50	30 / 42
*rlk2/rlk10*	0 / 91	13 / 80

A parallel assessment of nutrient-status and *NSP*-dependence on the regulation of symbiosis signaling genes in *M. truncatula* showed very little nutrient regulation of these signal transduction components (Fig. 6a), however, a number of genes encoding LysM*-*type receptor-like kinases showed nutrient-responsiveness, some of which were dependent on *NSP1* and/or *NSP2* (Fig. 6a). However, the known LCO receptors, *NFP* and *LYK3*, showed no upregulation in response to nutrient-starvation (Fig. 6a). We propose that other receptors, beyond those characterized, must explain nutrient responsive LCO perception in *M. truncatula*.

## Discussion

Plants regulate associations with beneficial microorganisms as a function of the levels of N and P in their surrounding environment^53–55^, likely due to the higher energetic costs associated with a symbiotic route for nutrient capture^16–18^. The mechanisms that allow this nutrient regulation of microbial engagement are only just beginning to be elucidated. We demonstrate that one mechanism for nutrient control of AMF associations is through the regulation of LCO perception and its ability to activate symbiosis signaling^22^. This signaling pathway is essential for AMF colonization^21^ and is particularly associated with the initial recognition of AMF at the root surface^11,56^. We show that the perception of LCOs in both *M. truncatula* and barley is strongly regulated by the availability of both N and P, with a combination of N- and P-starvation maximally activating LCO recognition. The regulation of CO perception and its ability to activate symbiosis signaling is much less responsive to nutrient availability, suggesting that a mechanism specific to LCO perception is likely the principle target for regulation. We propose transcriptional regulation of the LCO receptors, *RLK2* and *RLK10* in barley, provides such a mechanism. However, we also observe nutrient control of *SYMRK* and *CYCLOPS* expression and their upregulation by *NSP2* overexpression, pointing at additional targets for the nutrient regulation of this pathway. This may explain the qualitative differences we observe in the patterns of the calcium oscillations in plants grown under different nutrient regimes (Supplementary Fig. 2a, b). LCOs have recently been shown to be molecules produced by a range of fungal species, not only beneficial fungi^57^. However, LCO production is enhanced in AMF following the perception of plant-derived signals, such as strigolactones^8–10^, and this enhancement of LCO production has not been demonstrated in other species of fungi. We propose that even with LCOs produced by many fungi, there remain possible mechanisms for these molecules to act as symbiotic signals, either through the suite of decorated LCOs generated by specific fungi or by their enhanced production and delivery at the root surface. However, it is surprising that the *rlk2/rlk10* mutant that shows no LCO induction of symbiosis signaling and a greatly attenuated response to CO4, still shows 50% colonization by AMF, in contrast to the lack of colonization in symbiosis signaling mutants. This suggests that additional signals from AMF, yet to be defined, may contribute to the activation of symbiosis signaling in the host plant.

We demonstrate that nutrient regulation of LCO perception is a function of the transcription factors *NSP1* and *NSP2*. These genes regulate many components involved in the biosynthesis of apocarotenoids, in both *M. truncatula* and barley, suggesting that regulation of apocarotenoid biosynthesis is an important and conserved function of *NSPs* in plants. Numerous apocarotenoids have been associated with the AMF symbiosis, such as strigolactones, mycorradicins, blumenols, and zaxinones^48^. We demonstrate that *NSPs* are necessary and sufficient to activate strigolactone production, but the range of enzymes controlled by *NSPs* that are associated with apocarotenoid biosynthesis implies that these transcription factors are likely affecting a much broader range of apocarotenoid-derived small molecules. Our genetic analysis in *M. truncatula* is consistent with previous work in rice^36,37,47,50,51^ and demonstrates that strigolactones are primarily a signal exuded to the rhizosphere to promote interactions with the fungus, but their recognition by the plant via the receptor *D14* is irrelevant to AMF associations and may even be detrimental. In contrast, the karrikin-like receptor *D14L*, that is essential for AMF colonization^36^, is essential for both appropriate and *NSP2*-driven enhancement of AMF infection. In our work, we found that *D14L* is essential for responses to karrikins, but surprisingly was also essential for the 5DS induction of *RLK10* expression and resultant LCO perception. Villaécija-Aguilar et al.^58^ reported that root hair development in *d14* is still responsive to 5DS^58^, suggesting a *D14*-independent strigolactone receptor. D14L paralogs in pea are able to bind 5DS in vitro and hydrolyse strigolactone-like molecules^59^, raising the possibility that strigolactones or strigolactone-derived molecules^60^ may bind and activate D14L.

**Fig. 7:**
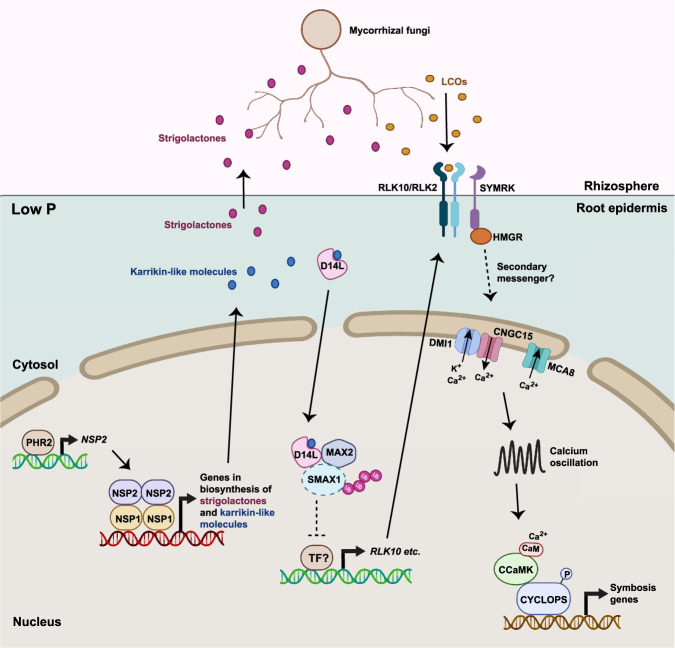
P-regulation of LCO production and perception, controlled by *NSP1/NSP2*. Under P-starvation conditions, *NSP2* expression is promoted by *PHR2*^32^, that functions with *NSP1* to promote gene expression associated with the biosynthesis of strigolactones and unknown karrikin-like molecules. Strigolactones are exuded to the rhizosphere to activate hyphal branching of mycorrhizal fungi and stimulate the production of fungal signals, including LCOs^8–10^. Karrikin-like signaling via D14L removes SMAX1 suppression, allowing the expression of the LCO receptor, *RLK10* in barley and *NFR5* in rice, allowing LCO recognition and activation of symbiosis signaling, triggering nuclear calcium oscillations and symbiotic gene expression. Whether strigolactones or strigolactone-derived molecules act as endogenous karrikin-like molecules needs to be further assessed. Taken together, *NSP1* and *NSP2* coordinate fungal and plant processes associated with LCO production and perception, to facilitate the AMF symbiosis. Created with BioRender.com.

We propose that *NSPs* likely control the production of small molecules required for the activation of D14L and subsequent suppression of SMAX1 (Fig. 7). Consistent with this hypothesis, we observe that *NSP2* overexpression in barley activates *RLK10/NFR5, SYMRK*, and *CYCLOPS*, genes that are also constitutively activated in the rice *smax1* mutant^37^. Thus *NSP* regulation during nutrient starvation coordinates external rhizopheric signaling to AMF via the production of strigolactones, with internal plant signaling promoting the expression of the LCO receptor and some components of the symbiosis signaling pathway. As such, *NSPs* can coordinate multiple processes preparing both AMF and the host plant for symbiosis (Fig. 7).

The transcription factors *PHR1* and *PHR2* are master regulators of the P-starvation response that have been demonstrated to also control associations with a range of microorganisms^1,31,32,61,62^. Of particular relevance is the very recent demonstrations that *PHRs* control the engagement with AMF, appearing to function at multiple levels during this association^31,32^. PHR2 directly binds the promoters of a range of genes in rice associated with cortical cells harboring arbuscules, with the regulation of proteins associated with nutrient exchange across the periarbuscular membrane^31^. In addition, PHR2 also directly regulates the expression of *CERK1* and *SYMRK* in rice, that are associated with the perception of COs and the symbiosis signaling pathway, suggesting an additional role for *PHR2* at the root surface for initial perception of AMF^32^. While the expression of the LCO receptor *NFR5*^14,34^ is affected by the *phr2* mutant, this gene is not directly regulated by *PHR2*^32^. Instead, *NSP2* is a direct target of *PHR2* and we propose that the regulation of the LCO receptor by *PHR2* is through the regulation of *NSP2*, as demonstrated here. This implies that *PHR2* can regulate the symbiosis signaling pathway via two mechanisms: direct control of *CERK1* and *SYMRK* and indirect control of *NFR5, SYMRK*, and *CYCLOPS* through the promotion of *NSP2* expression. Overexpression of both *NSP2* (this study) and *PHR2*^31,32^ is sufficient to partially override P-suppression of AMF colonization, with the relative levels of impact comparable. This further supports a linear relationship between *PHR2* and *NSP2* function, at least for the regulation of AMF colonization at the root surface. *NSP2* overexpression does not induce any of the *PHR2-*induced genes associated with the arbuscule^31^, making it difficult to judge whether *NSPs* also function downstream of *PHR2* at this late stage of the AMF association. However, *nsp* mutants of barley show strong reductions in numbers of arbuscules (Fig. 3f), implying a possible role for *NSPs* also during arbuscule development.

Nutrient regulation of LCO recognition by *NSPs* is conserved between *M. truncatula* and barley, implying a process that probably evolved early in the plant kingdom, likely alongside the evolution of symbiosis signaling^63^, to support AMF symbioses. Within the last 100 million years, legumes augmented their symbiotic repertoire with the addition of associations with N-fixing bacteria. During this evolutionary process, legumes utilized the preexisting symbiosis signaling pathway to allow recognition of N-fixing bacteria^64^. Our work reveals that whereas both *M. truncatula* and barley can activate symbiosis signaling via equivalent signaling molecules, including LCO Nod factors produced by N-fixing bacteria, legumes appear to have expanded their specificity of LCO recognition, showing exquisite sensitivity for appropriately decorated LCOs generated by their N-fixing symbiont. This is consistent with the specific decorations on LCOs providing host legume specificity for N-fixing symbionts^65^. *NSP1* and *NSP2* have essential roles in nodulation^38,39^, and this function appears to be during nodule establishment, downstream of both symbiosis signaling and cytokinin signaling^23,66^. This suggests additional functions for *NSP1* and *NSP2* during nodulation, that may be unrelated to their functions in preparation of the plant for AMF colonization. An expansion for *NSP2* function may have been facilitated by the duplication of this gene in legumes, with genetic redundancy between *NSP2* and *NSP2L* for AMF associations in *M. truncatula*, but no such genetic redundancy for *NSP2* during nodulation^67^. It remains unclear whether *NSP2* function during nodulation is associated with apocarotenoid biosynthesis, however, the fact that neither strigolactones or *D14L* have positive functions during nodule establishment, points at quite different targets and modes of action for *NSPs* during nodule organogenesis.

Achieving sustainable productivity in agriculture necessitates a reduction in the global dependence on inorganic fertilizers and this in part could be achieved through better use of beneficial microbial associations. Our work demonstrates a mechanism to enhance colonization of AMF, in particular under suppressive P-conditions, using genes that have symbiotic specific functions, that is preferable to using genes such as *PHR2* that have very broad effects on plant function. Enhancing AMF associations in agriculture has the potential to improve the efficiency of nutrient capture, reducing loss of these nutrients to the environment^68^. Furthermore, we have also demonstrated major differences between legumes and cereals in the stringency of LCO recognition, a process already well understood^69^. This is likely an important engineering target for generating N-fixing cereals. Agricultural processes that rely more heavily on beneficial microbial nutrient delivery as oppose to the application of inorganic fertilizers, have the potential to deliver more sustainable and equitable global food production.

## Methods

### Plant materials

Barley (*Hordeum vulgare* L. cv. Golden Promise), wheat (*Triticum aestivum* L. cv Fielder), maize (Zea mays L.), and rice (*Oryza sativa* cv. Nipponbare) were transformed with the calcium reporter cameleon YC3.6 using *Agrobacterium tumefaciens-*mediated transformation^70–72^. Barley *symrk*, *ccamk*, *cyclops*, *ram1*, *nsp1*, *nsp2*, and *d14l* mutants were generated with CRISPR/Cas9^73^. YC3.6 was introduced into the barley *symrk-1* and rice *pollux*^36^ and *d14l*^37^ through *Agrobacterium-*mediated transformation^70^. Individual T2 lines showing strong expression of YC3.6 were selected for calcium analysis. *Medicago truncatula* A17 was used as the wild type of *nsp1-1*, *nsp2-2*, and *nsp1/nsp2* mutants. YC3.6 was incorporated into *nsp1/nsp2* mutant by crossing with an existing stable YC3.6 line in A17 background. *Medicago truncatula* R108 was used as the control for the *Tnt1* insertion mutants *nsp1-3* (NF9220), *nsp2-4* (NF10950), *nsp2l-1* (NF17492), *ccd7-1* (NF1485), *ccd8a-2* (NF18323), *ccd8a-3* (NF111036), *ccd8a-3* (NF111036), *d14-1* (NF18262), *d14la-1* (NF13623), *d14lb-1* (NF5873), *max2-1* (NF18521), and *smax1-1* (NF4373). All *Tnt1* lines were ordered from the Samuel Roberts Noble Foundation collection^74^. The primers for genotyping of these *Tnt1* mutants are listed in Table S4.

### Plant growth conditions

Barley, wheat, and maize seeds were treated with 70% ethanol for 2 minutes, followed with three times wash in sterile water. The seeds were then surfaced sterilized by 5% sodium hypochlorite solution for 4 minutes and rinsed with sterile water 4–5 times. The sterilized seeds were plated on 1% water agar plates and imbibed at 4 °C for 3 days and then germinated in the dark at 22 °C for 2–3 days. Seedlings were cultivated in a 1:1 mixture of sterilized terragreen (Oil‐dry UK ltd) and sharp sand (BB Minerals) in growth chambers at 20 °C under 16 h light/8 h dark conditions with 80% relative humidity.

Rice seeds were surface-sterilized briefly in 70% (v/v) ethanol, then for 20 min in 3% (v/v) sodium hypochlorite, and finally rinsed three times in sterile water. Imbibed seeds were germinated on 0.6% (w/v) bactoagar in the dark at 30 °C for 3-7 days. Seedlings were transferred into cones containing sterile quartz sand and cultivated in growth chambers at 28 °C/20 °C, 12 h light/12 h dark cycle with 60% relative humidity.

*M. truncatula* seeds were scarified with sandpaper and surface-sterilized in 10% sodium hypochlorite solution for 6 mins and rinsed 4–5 times in sterile water. The seeds planted on 1% water agar were imbibed at 4 °C for 3 days and germinated overnight in the dark at 22 °C. Seedlings were cultivated in growth chambers at 20 °C under 16 h light/8 h dark conditions with 80% relative humidity.

### Nuclear calcium imaging

For barley, wheat and maize calcium analyses in Figs. 1a and 2b, plants were grown in 1:1 mixture of sterilized Terragreen and sharp sand mix and watered with sterile water only for 25 days. For rice calcium analyses in Fig. 2a, plants were grown in cones containing sterile quartz sand and watered three times weekly for the first week, and subsequently watered once a week and fertilized twice a week with half Hoagland solution (25 μM PO_4_^3-^) until plants were five weeks old. These growth conditions were previously demonstrated to promote efficient mycorrhizal colonization^21^. For calcium analyses in Fig. 1b, c, *M. truncatula* and barley seedlings were grown for 10 days on BNM^75^ plates without aminoethoxyvinylglycine (AVG, Sigma-Aldrich) until lateral roots emerged.

For nutrient treatments on *M. truncatula* and barley, seedlings were grown for 16 days on modified BNM plates with the concentrations of N and P as: -N-P, no NO_3_^−^ and no PO_4_^3−^; −N + P, no NO_3_^−^ and 0.5 mM PO_4_^3−^; + N-P, 5 mM NO_3_^-^ and no PO_4_^3−^; + N + P, 5 mM NO_3_^−^ and 0.5 mM PO_4_^3−^. The concentration of P used reflects that which was sufficient to significantly suppress AMF colonization in *M. truncatula* and barley (Fig. 4a, b).

For the pretreatment of strigolactone and karrikin on barley, *M. truncatula* and wheat, seedlings were grown on BNM plates (−N + P, no NO_3_^−^ and 0.5 mM PO_4_^3−^) for 5 days (barley and wheat) or 10 days (*M. truncatula*) until lateral roots emerged, and then were treated in BNM liquid medium with 0.1 μM or 1 μM (±)−5-deoxystrigol (Chiralix), a 0.1 μM or 1 μM mixture of karrikin 1 and karrikin 2 (Chiralix) or 1 μM GR24 (Chiralix), using BNM liquid adding the same volume of acetone (Fisher Chemical) as a control. Following the treatment of lateral roots with different concentrations of COs and LCOs, the measurement of calcium oscillations was performed using an inverted epifluorescence microscope (model TE2000; Nikon). Calcium recordings were collected and analyzed as described previously^11^.

### Generation of barley CRISPR mutants and barley transgenic lines

The methods for creating barley CRISPR mutants are as described^73^. The specific target sequences of the guide RNAs, and the mutations present in each mutant plant, are listed in Supplementary Table 5. *Hordeum vulgare* cv. Golden Promise wild type or YC3.6 lines were transformed as previously described^70^ with the constructs (L2 plasmids) listed in Supplementary Table 3. Briefly, the constructs were transformed into *Agrobacterium tumefaciens* strain AGL1. Immature barley seeds (with embryo size around 1.5 mm in diameter) were harvested and sterilized with 70% ethanol for 2 minutes, followed by 3 washes in sterile water. The seeds were then sterilized with 5% sodium hypochlorite solution for 4 minutes and rinsed with sterile water 4–5 times. Immature embryos were isolated using fine forceps in a laminar flow hood and the embryonic axis was removed from the scutellum and discarded. They were then cultured on Callus induction (CI) plates^70^ at 23–24 °C in the dark overnight. The embryos were treated with *Agrobacterium* inoculum and transferred to new CI plates for co-cultivation. After 3 days, the embryos were transferred to CI plates with antibiotics: Timentin, to remove the *Agrobacterium* and hygromycin, for selection of transgenic cells. The following culture at 23–24 °C in the dark for 2 weeks, embryos were transferred to fresh CI plates and cultured under the same conditions for another 4 weeks to induce callus formation, with subculture to fresh plates after 2 weeks. Calli were then transferred to Transition medium under low light for 2 weeks. Calli that developed shoots were transferred to Regeneration plates^70^ until small plantlets formed. Individual plantlets were separated and transferred to rooting tubes. Once plantlets reached the top of the tubes and had good root systems, they were transferred to soil for growth to maturity.

### Mycorrhizal inoculation

For mycorrhization assays, the barley seedlings were inoculated with 5% or 10% (w/w) crude inoculum (produced on *Tagetes multiflora*) of *R. irregularis* and grown in a cone system at 28 °C/20 °C,12 h light/12 h dark cycle with 60% humidity. Plants were watered with water only for the first 1–2 weeks, then were nurtured with half-strength Hoagland solution containing 10 μM PO_4_^3−^ twice a week. For nutrient treatments on barley *NSP* overexpression lines, half-strength Hoagland solutions containing a range of P concentrations (10 μM, 500 μM, and 1 mM PO_4_^3−^) were given twice a week repeatedly. The inoculated roots were harvested and stained by Trypan blue^76^. Stained roots were mounted on slides (ten root pieces per slide), and fungal colonization was quantified at 10 representative random points per root piece.

*M. truncatula* plants were grown in 4 x 4 x 4.5 cm^3^ pots containing sharp sand and 200 *R. irregularis* spores. *R. irregularis* spores were ordered from Premier Tech (Québec, Canada). Plants were fertilized twice per week with modified BNM liquid medium (5 mM NO_3_^−^ and 10 μM PO_4_^3−^) for 3 weeks. For nutrient treatments on hairy-root transformed *NSP*-overexpression plants, modified BNM solutions containing low and high P (10 μM and 500 μM PO_4_^3−^, 5 mM NO_3_^−^) were given twice a week until harvesting at 5 weeks post inoculation. The harvest roots were stained with an ink-vinegar solution^77^. The roots were cut into 1 cm segments and pre‐cleared by incubation in 10% (w/v) KOH at 95 °C for 10 min. Roots were rinsed 3 times with distilled water and stained with a solution containing 5% ink and 5% acetic acid at 95 °C for 3–5 min. Roots were de‐stained in water for 1–2 days, and fungal structures were quantified using the gridline intersect method^78^. Fungal colonization was visualized using a Keyence digital microscope (Keyence VHX-5000).

### Plasmid construction

The plasmids were all constructed using the Golden Gate cloning system^79,80^. The gene sequences were domesticated and synthesized, and then cloned into level 0 vector pMS or pMK (GeneArt, Thermo Fisher Scientific). The assemblies of level 1 and level 2 plasmids are listed in Table S3. All the plasmids used in this study are held for distribution in the ENSA project collection (https://www.ensa.ac.uk/). *pMtD27::GUS* transcriptional reporter was generated using 1045 bp of the *MtD27* promotor and cloned in modified pCAMBIA2200 binary vector^81^.

### Hairy root transformation

The hairy root transformation assay was performed as described^82^. For Fig. 4a, *M. truncatula* seedlings were transformed via *A. rhizogenes* strain ARqua1 mediated hairy root transformation with either an empty vector carrying a RUBY reporter^83^ or vectors with overexpression of *NSP* genes (Supplementary Table 3). Three weeks after transformation, plants with *RUBY*-expressing roots were selected for further assays.

For Supplementary Fig. 8b, c, *M. truncatula* seedlings were transformed via *A. rhizogenes* strain AR1193 mediated hairy root transformation with the vectors containing a *DsRed* reporter. The plants were grown on modFP plates for four weeks after hairy-root transformation. The transformed roots were screened using an Axiozoom V16 (Zeiss) or DMR/MZFLIII (Leica) microscope to visualize the transformation marker DsRed. The positive roots were harvested for GUS staining or gene expression analyses.

### RNA extraction and RT-qPCR

Barley seedlings were grown in sterilized sharp sand for 21 days, fertilized twice a week with modified BNM liquid with the concentrations of N and P as: −N−P, no NO_3_^−^ and no PO_4_^3−^; −N + P, no NO_3_^−^ and 0.5 mM PO_4_^3−^; + N-P, 5 mM NO_3_^−^ and no PO_4_^3−^; + N + P, 5 mM NO_3_^−^ and 0.5 mM PO_4_^3−^. For the pretreatment of strigolactone and karrikin on barley, seedlings were grown on modified BNM plates (+N + P, 3 mM NO_3_^-^ and 0.5 mM PO_4_^3−^) for 3 days, and then were treated on modified BNM solid medium containing 1 μM (±)-5-deoxystrigol (Chiralix) and a 1 μM mixture of karrikin 1 and karrikin 2 (Chiralix) for 2 days.

*M. truncatula* seedlings of A17, *nsp1-1*, and *nsp2-2* were grown on modified BNM plates for 15 days with the concentrations of N and P as: −N−P, no NO_3_^−^ and no PO_4_^3−^; −N + P, no NO_3_^−^ and 3.75 mM PO_4_^3−^; + N−P, 5 mM NO_3_^−^ and no PO_4_^3−^; + N + P, 5 mM NO_3_^−^ and 3.75 mM PO_4_^3^. The concentrations of P used reflect those calculated as being necessary for suppression of AMF colonization in *M. truncatula*^55^. The *A. rhizogenes* transformed *M. truncatula* roots with *NSP* overexpression were transferred into sterilized sharp sand after the selection by a RUBY reporter and watered with modified BNM liquid (+N + P, 5 mM NO_3_^−^ and 0.5 mM PO_4_^3−^) twice a week for three weeks. The roots were harvested and stored at −80 °C for RNA extraction.

Barley total RNA was extracted using the Spectrum™ Plant Total RNA kit (Sigma-Aldrich) and *M. truncatula* total RNA was isolated with the RNeasy PlantMini Kit (Qiagen), following the manufacturer’s instructions. RNA concentration was determined by NanoDrop One (Thermo Scientific). 1μg of total RNA was used for cDNA synthesis with the SensiFAST cDNA Synthesis Kit (Bioline). Real-time quantitative PCR (RT-qPCR) was performed using SensiFAST SYBR No-ROX Kit and semi-quantitative RT-PCR using GoTaq Green Master Mix (Promega). The primers used for gene expression analysis are listed in Supplementary Table 4.

### RNA-seq and data analysis

Large scale transcriptomic RNA sequencing (RNA-seq) was performed by GeneWiz (Leipzig, Germany) and Novogene (Cambridge, United Kingdom). RNA samples were sent for library preparation and sequencing with Illumina NovaSeq Next Generation Sequencing system as paired-end 2 × 150 bp reads. Raw fastq files generated from sequencing were further subjected to quality controls with FastQC software^84^ and then mapped to the *Medicago truncatula* reference genome 4.0 (Mt4.0v1)^85^ from Phytozome database^86^ or the *Hordeum vulgare* cv. Golden Promise reference genome^63,87^, using STAR software^88^. The counts and FPKM (Fragments per kilobase per million mapped reads) values were calculated with featureCounts in R package Rsubread^89^. Non-metric multidimensional scaling was exploited to account for outliers^90^. Genes that showed low expression throughout all samples were removed by measuring CPM (counts per million) values using R package edgeR^91^ to filter out genes with CPM values corresponding to fewer than 10 raw counts. TMM expression values were obtained using R package edgeR^91^ to conduct TMM normalization of the library sizes and output CPM values normalized by the resultant effective library sizes. Differentially expressed genes (DEGs) were identified by pairwise comparisons of raw counts of nutrient replete versus depleted treatments or wildtype versus mutant/transgenic lines for nutrient and genotypic effects, respectively, using the R package DESeq2^92^ with a false discovery rate (FDR) corrected *p*-value more significant than 0.05. The heatmaps of differential expression and hierarchical clustering were plotted with Python package Seaborn^93^, with hierarchical clustering applying the “correlation” metric and displaying DEGs with a threshold of absolute fold change over 1.5. Synonyms and descriptions of *Medicago truncatula* DEGs were obtained as described previously^94^. Putative synonyms and descriptions of *Hordeum vulgare* DEGs were assigned by employing the Orthofinder tool^95,96^ to infer orthology relationships to genes from other annotated genomes, followed by manual curation of descriptions. For a more robust inference, OrthoFinder analysis was run with default parameters, including four proteomes: *Oryza sativa* v7.0^97^, *Arabidopsis thaliana* TAIR10^98^, *Medicago truncatula* Mt4.0v1^85^, and *Hordeum vulgare* cv. Golden Promise v1r1^63^.

### Generation of phylogenetic trees

Homologs of genes of interest were searched against the genomes of *Arabidopsis thaliana* Araport11^99^, *Fragaria vesca* v4.0^15^, *Medicago truncatula* Mt4.0v1^85^, *Lotus japonicus* Gifu v1.2^100^, *Oryza sativa* v7.0^97^, *Zea mays* PH207 v1.1^101^, and *Hordeum vulgare*^63^. Searches were performed with the tBLASTn algorithm v2.10.1 + ^102^ with the protein sequences of *M. truncatula* NSP1 and NSP2, and *Arabidopsis* CCD8, KAI2, and SMAX1 as query. *LysM-RLK* homologs were found by using previously identified *Medicago* genes from the family^103^ as query, combined with barley genes identified as containing both LysM (PF01476) and protein kinase (PF00069) domains from the Pfam database^104^ using HMMR 3.3.2^105^. Coding sequences of obtained hits were aligned with MAFFT v7^106^ and phylogenetic tree construction by maximum likelihood was performed using IQ-TREE with 1000 replicates of ultrafast bootstrap^107^. Trees were visualized and annotated on the Interactive Tree Of Life (iTOL) platform, with clades that included the gene(s) of interest pruned from the tree and bootstrap information displayed as circles^108^.

### Measurement of strigolactones

Barley seedlings were grown in sterilized sand for six weeks, watered twice a week with a modified half-strength Hoaglands nutrient solution [2.4 mM KNO_3_, 1.6 mM Ca(NO_3_)_2_, 0.5 mM KH_2_PO_4_, 0.8 mM MgSO_4_, 0.18 mM FeSO_4_, 0.1 mM Na_2_EDTA, 4.5 µM MnCl_2_, 23 µM H_3_BO_3_, 0.3 µM CuSO_4_, 1.5 µM ZnCl_2_, and 0.1 µM Na_2_MoO_4_] (high P, HP). P starvation stress was induced by reducing the amount available PO_4_^-^ to 0.02 mM KH_2_PO_4_ (low P, LP). After six weeks, all pots were rinsed with 1 L half-strength Hoaglands nutrient solution (either HP or LP), and grown for an additional week under these nutrient conditions before harvesting the roots. Strigolactones were extracted as previously described^109,110^, with some modifications. Extracts were evaporated to dryness, taken up in 100% hexane, and loaded on pre-equilibrated Silica gel SPE (Grace Pure^TM^ SPE Silica 100 mg/1 mL) columns. Samples were then eluted with 1 ml of hexane:ethyl acetate (1:9) and evaporated to dryness. The residue was dissolved in 200 μl of 20% acetonitrile/water (v/v) spiked with 10^−^^6 ^M GR24. Samples were filtered using a 0.2 µm Mini spin column (BGB Analytik Benelux B.V.). and stored for maximum 24 h at 4 ˚C before UPLC-MS/MS analysis. SLs were analyzed by comparing retention times and mass transitions with a 5-deoxystrigol standard using a Waters Xevo TQ mass spectrometer equipped with an electrospray-ionization source and coupled to a Waters Acquity Ultraperformance LC system as previously described^43^ with some modifications^111–113^. GR24 served as an internal standard that was added before analysis. Detection and quantification of SLs were performed with six biological replicates.

### Nodulation assay

*M. truncatula* plants were grown in 4 x 4 x 4.5 cm^3^ pots containing 1:1 mix of sterile terragreen/sharp sand for seven days and inoculated with 5 mL *S. meliloti* 2011 (OD_600_: 0.02) in each pot. Nodules were harvested for quantification at two weeks post inoculation.

### GUS staining

For histochemical GUS analysis, root tissues of *pMtD27::GUS* hairy roots were soaked in GUS staining solution (100 mM sodium phosphate buffer, pH 7.2, 10 mM EDTA, 0.1 % Triton X-100, 0.3 mg/ml X-Gluc) under vacuum for 15 min. Root tissues were then incubated 2–3 h in GUS staining solution at 37 °C. Staining is stopped at the same time for WT and *nsp1-1* mutant by washing the roots with water, then roots are fixed in 50% ethanol for long-term storage and imagining using Axiozoom V16 (Zeiss) under brightfield.

### Reporting summary

Further information on research design is available in the Nature Research Reporting Summary linked to this article.

## Supplementary information

Supplementary Information

Reporting Summary

## Data Availability

The raw data of RNA-sequencing have been deposited in the National Center for Biotechnology Information, Gene Expression Omnibus (GEO), with accession code GSE214698. Any additional information required to reanalyze the data reported in this study is available from the lead contact upon request. Source data are provided with this paper.

## Source data

Source data
