# Supplementary material for: Nutrient regulation of lipochitooligosaccharide recognition in plants via NSP1 and NSP2
Source: Nat Commun. 2022 Oct 28;13:6421. doi: 10.1038/s41467-022-33908-3 (PMC9616857; doi:10.1038/s41467-022-33908-3)
Supplement: Supplementary file 1 — Supplementary Information [file 41467_2022_33908_MOESM1_ESM.pdf]

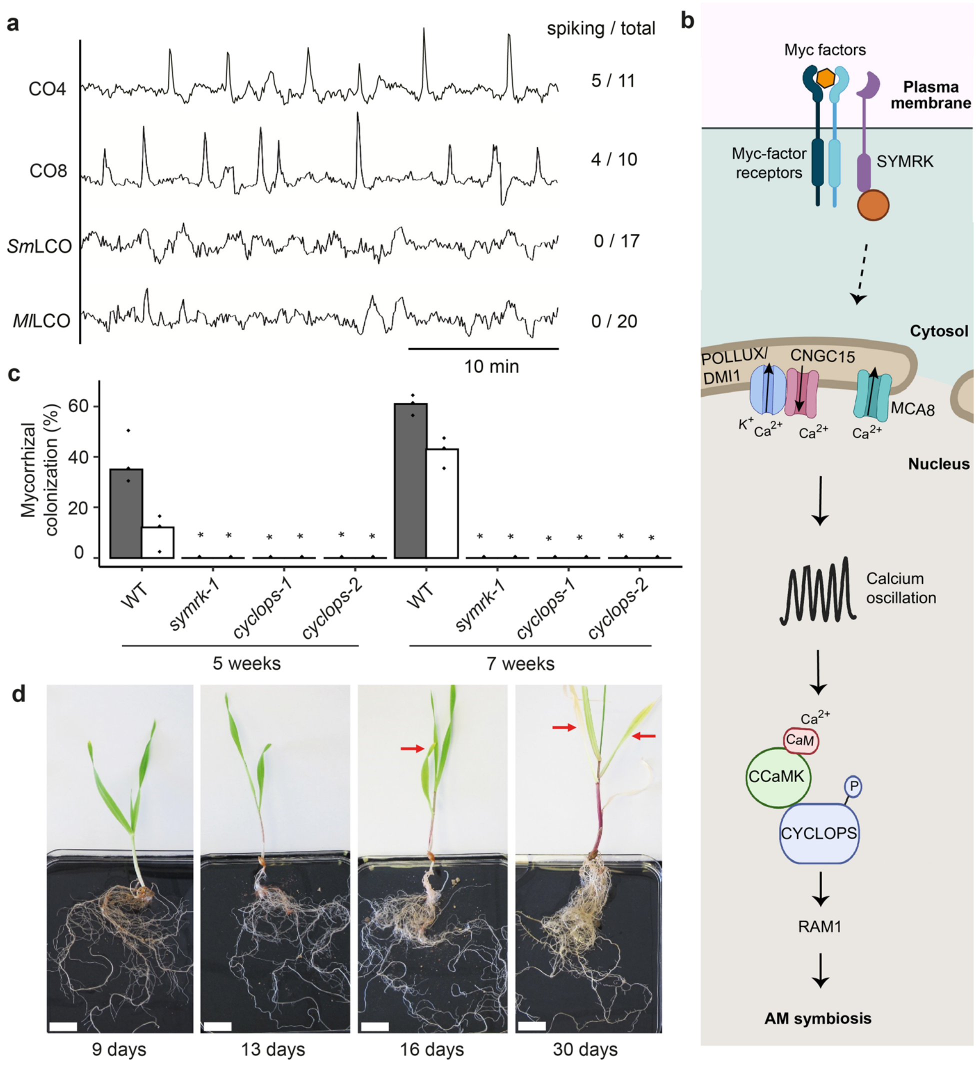

Supplementary Figure 1. CO and LCO response in barley. a Responses of barley atrichoblasts to COs and LCOs at early developmental stages. Representative traces in atrichoblasts of 5-day-old lateral roots under nutrient limited conditions, responding to treatment of 10^{-8} M CO4, 10^{-8} M CO8, 10^{-7} M *Sm*LCO and 10^{-7} M *Ml*LCO. Numbers indicate cells responding compared to total cells analyzed. **b** A simplified signaling pathway of arbuscular mycorrhizal (AM) symbiosis. The Myc factors (Myc-LCOs and Myc-COs) secreted by AMF are perceived by the Myc-factor receptors (LysM receptor kinases) and the receptor-like kinase SYMRK in the plasma membrane of the plant cells. Then an unknown secondary messenger transduces the signal into the nucleus, causing nuclear calcium oscillations which are supported by the channels POLLUX/DMI, CNGC15 and MCA8 in the nuclear membrane. The nuclear calcium signal is decoded by CCaMK via association with calmodulin (CaM) and calcium, inducing the phosphorylation of CYCLOPS. The CCaMK-CYCLOPS complex promotes the expression of transcription factor *RAM1*, which is a major regulator of AM symbiosis. **c** Related to Fig. 1d, showing AMF colonization measured at 5- and 7-weeks post inoculation of barley wild type (WT), *symrk* and *cyclops* mutants. **d** Images of barley plants grown at indicated time points under nutrient limited conditions. Note in 16- and 30-day old plants the leaves begin to senesce (arrows) and anthocyanin accumulates in the stem, timepoints when LCO recognition is observed in the roots. Scale bars = 2 cm.

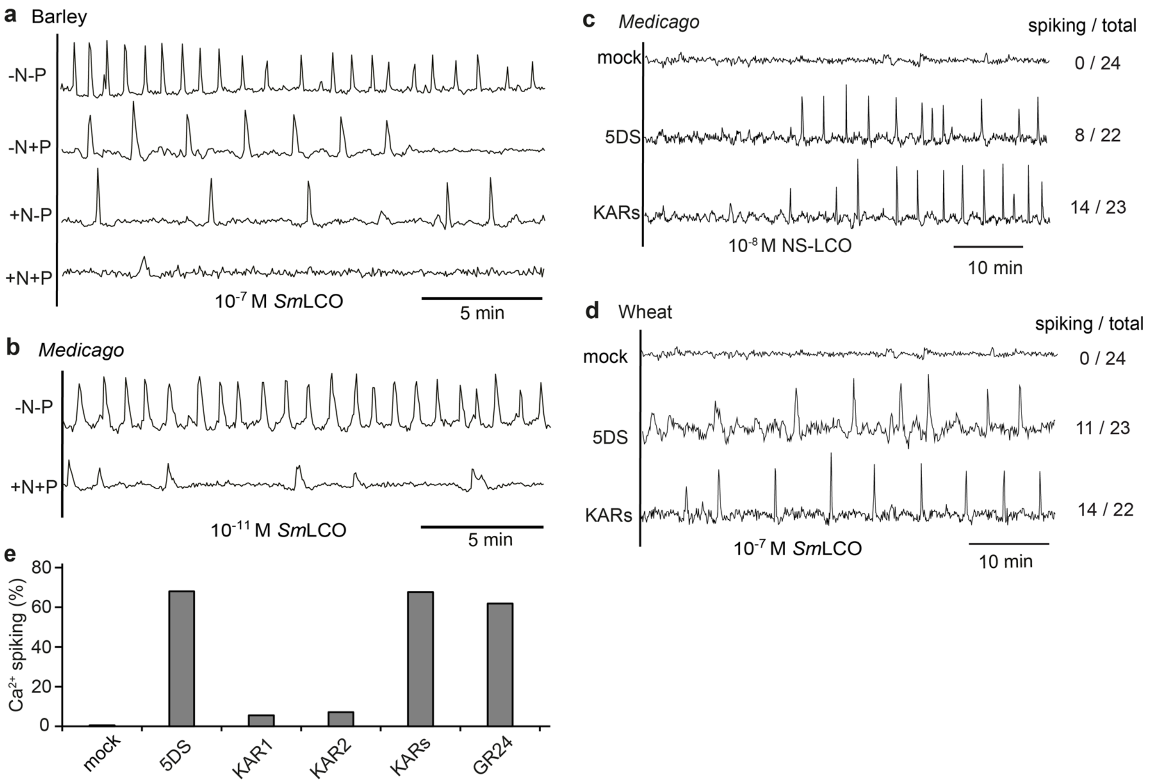

Supplementary Figure 2. Nutrient starvation and pretreatment of strigolactones and karrikins enhance calcium oscillations. a Related to Fig. 3a, the representative calcium traces in atrichoblasts of barley lateral roots grown under different nutrient regimes for 16 days, responding to 10^{-7} M *Sm*LCO. **b** Related to Fig. 3b, the calcium traces in atrichoblasts of *M. truncatula* lateral roots grown under nutrient depleted and replete conditions, responding to 10^{-11} M *Sm*LCO. **c and d** The calcium traces of atrichoblasts of *M. truncatula* responding to NS-LCO (**c**) and wheat lateral roots responding to 10^{-7}M *Sm*LCO (**d**) after pretreatment of control buffer (mock), 1 µM 5DS and a mixture of 1 µM KARs for 12 hrs. Plants were grown on +P-N medium for 10 days (*M. truncatula*) or 5 days (wheat) before pretreatment. Numbers indicate cells responding compared to total cells analyzed. **e** The percentage of atrichoblasts of barley lateral roots with calcium oscillations after pretreatment of mock, 1 µM 5DS, 1µM karrikin 1, 1µM karrikin 2, a mixture of 1 µM karrikin 1 and karrikin 2 (KARs) and 1 µM GR24. The plants were grown on +P-N medium with 100nM AVG for three days.

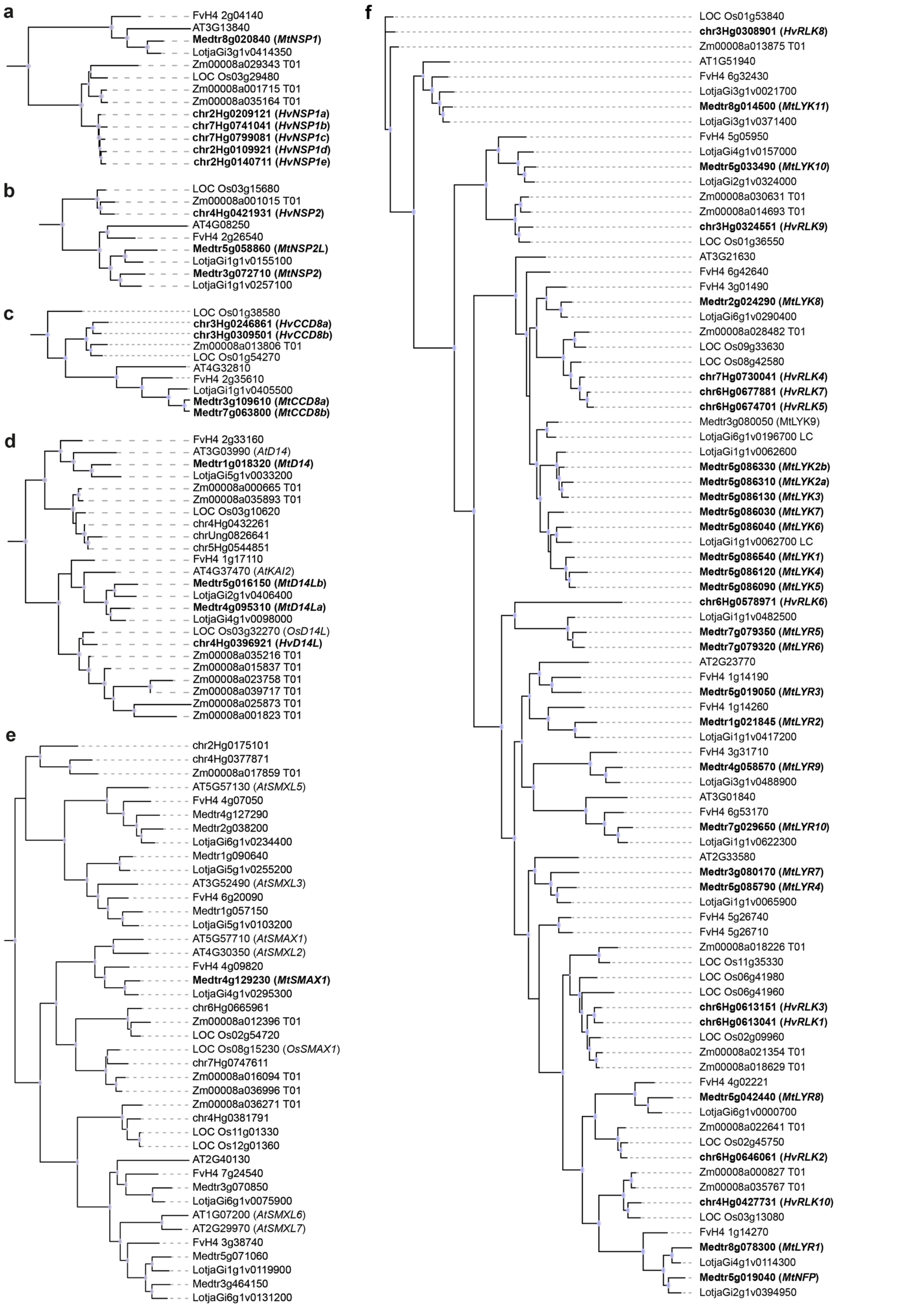

Supplementary Figure 3. Phylogentic trees of gene families, related to Figure 4, 6 and 7. Phylogentic trees represent the evolutionary relationship among *NSP1* (**a**), *NSP2* (**b**), *CCD8* (**c**), *D14/D14L* (**d**), *SMAX-LIKE* (**e**) and *LysM-RLK* (**f**) sequences from *Medicago truncatula*, *Hordeum vulgare*, *Lotus japonicus*, *Arabidopsis thaliana*, *Oryza sativa*, *Zea mays* and *Fragaria vesca*. The genes in *M. truncatula* and/or barley (*Hordeum vulgare*) are highlighted in bold. Bootstrap information is displayed as circles.

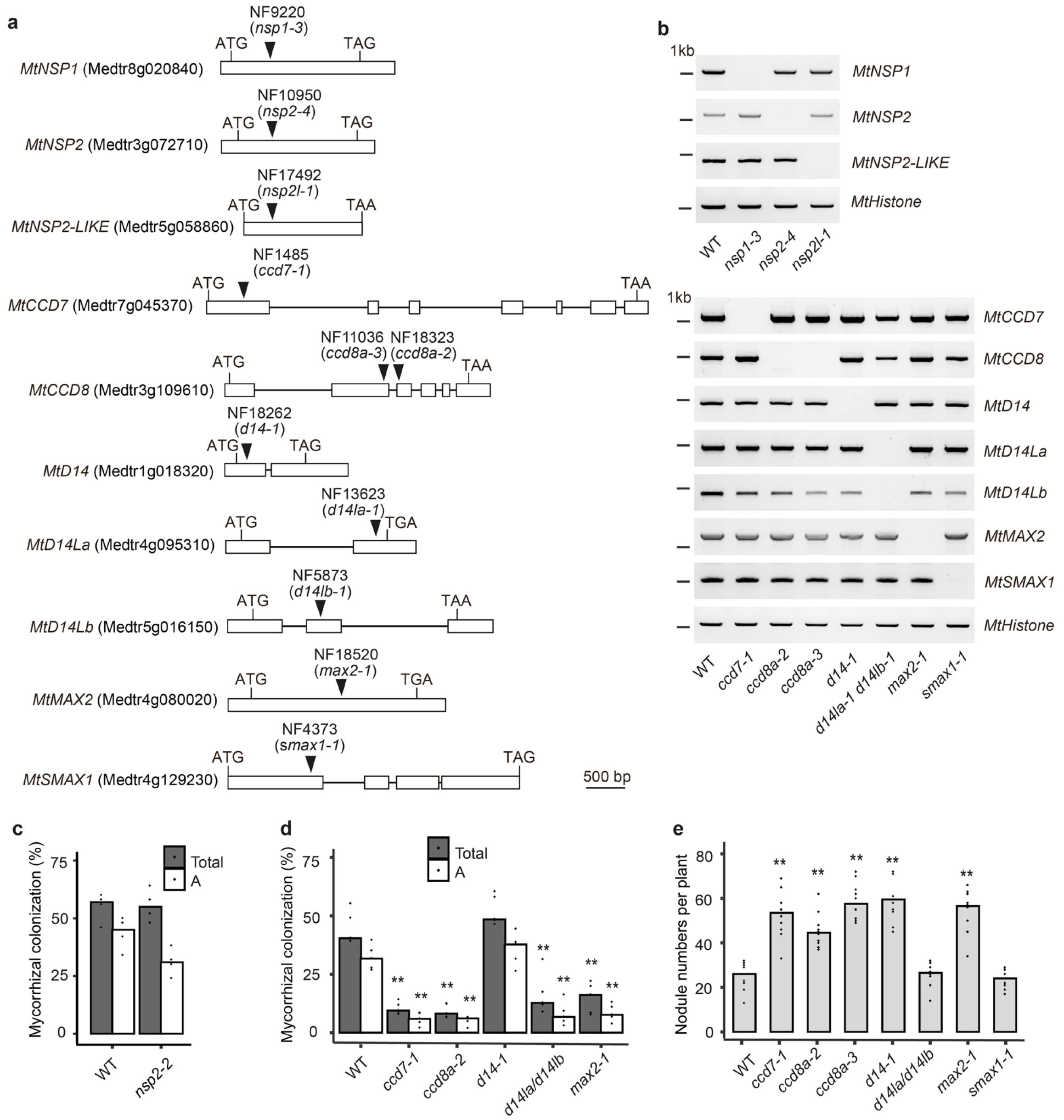

Supplementary Figure 4. Mycorrhizal colonization and nodulation of *M. truncatula* mutants. a The position of *Tnt1* insertion is annotated in the structure of each gene. **b** Semi-quantitative RT-PCR showing the transcript of each gene in the corresponding mutants, using *MtHistone* as a loading control. The assay was repeated three times with similar results. **c** Fungal colonization of wild type (A17) and *nsp2-2* mutants under P-deficient conditions, measured at 3 weeks post inoculation. **d** Related to Fig. 5c, a repeat of *R. irregularis* colonization in the critical mutants. n=5 biologically independent samples. Total: total colonization; A: arbuscules. **e** Nodulation of *M. truncatula* in strigolactone biosynthesis and strigolactone/karrikin signaling mutants under N-limited conditions. Number of nodules formed per plant was measured at 2 weeks post inoculation. n=10 biologically independent plants. For statistical analysis a one-sided Wilcoxon test was performed. **: $p<0.01$.

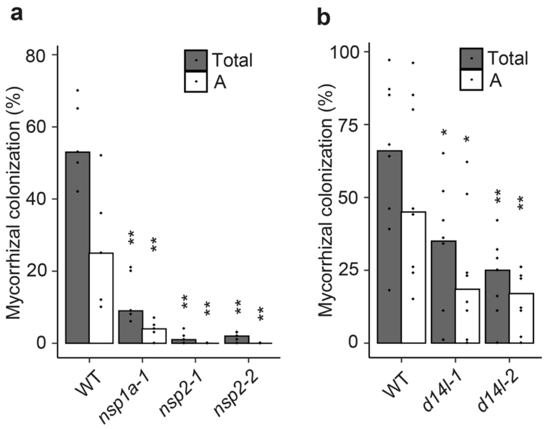

Supplementary Figure 5. Mycorrhizal colonization of barley mutants. a A repeat assay of Fig. 3f, showing root-length colonization of barley wild type, *nsp1a* and *nsp2* mutants at 7 weeks post inoculation. n=5 biologically independent samples. **b** Related to Fig. 5d, Root-length colonization at 9 weeks post inoculation in barley wild type and *d14l* mutant roots under P deficient conditions. n=8 biologically independent samples. Total: total colonization; A: arbuscules. *p*-values for colonization levels were determined by a one-sided Wilcoxon test. **: p<0.01; *: 0.01<p<0.05.

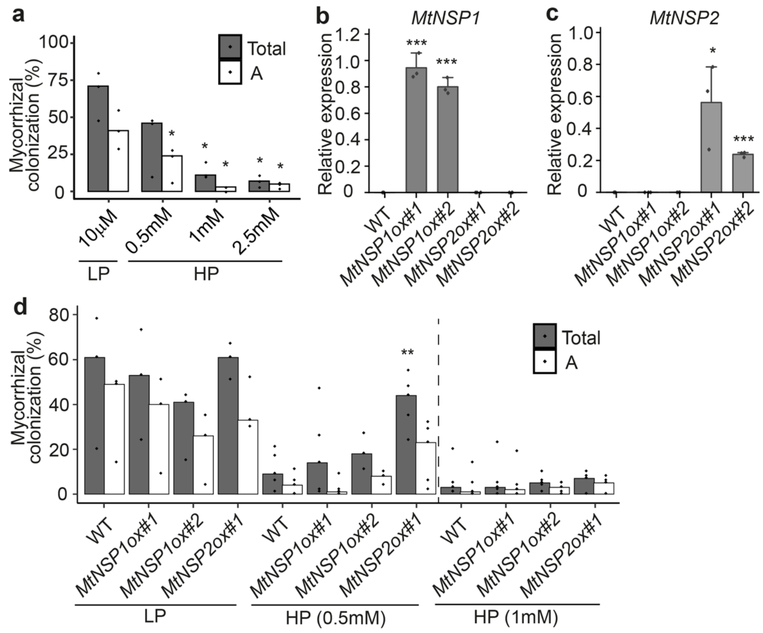

Supplementary Figure 6. Overexpression of *NSP2* in barley restored mycorrhization supressed by high P. a Mycorrhizal colonization levels of barley wild-type roots grown under P gradients, assessed at 7 weeks post inoculation. n=3 biologically independent plants. Total: total colonization; A: arbuscules. For statistical analysis a one-sided Wilcoxon test was performed. **: p<0.01. **b and c** Levels of *MtNSP1* (**b**) and *MtNSP2* (**c**) transcripts in barley roots transformed with overexpression of respective genes. The plants were grown under +P+N for three weeks. n=3 biologically independent samples. ±s.e.m. **: p<0.001, measured using a Student's t-test (one-tailed, two-sample equal variance). **d** A repeat experiment showing root-length colonization of barley roots overexpressing *MtNSP1* and *MtNSP2*. The plants were inoculated with *R. irregularis* and grown under different P levels for 5 weeks. n=3-5 biologically independent plants. Total: total colonization; A: arbuscules. p-values were determined by a one-sided Wilcoxon test. **: p<0.01.

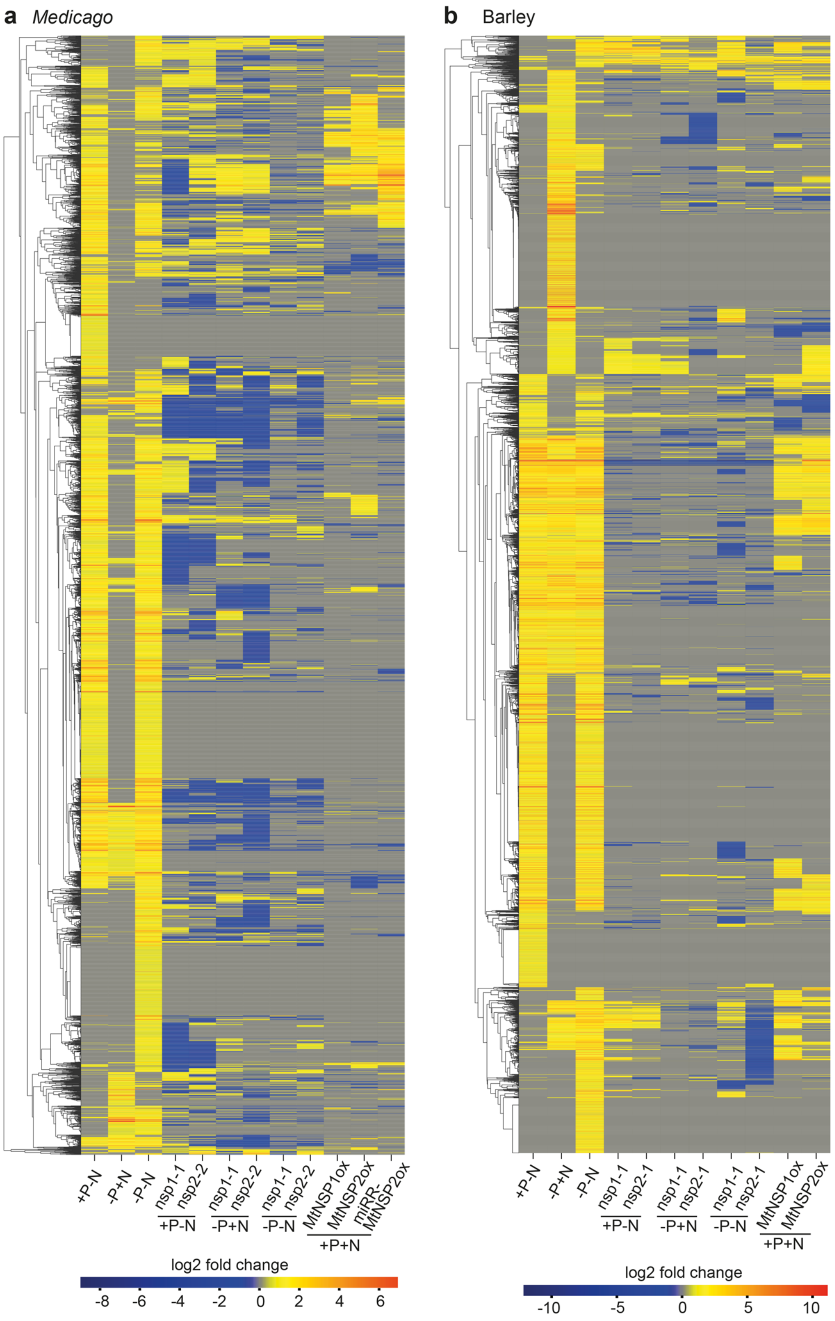

Supplementary Figure 7. N and/or P starvation induces a subset of genes regulated by *NSP1* and *NSP2*.

Heatmaps showing the *M. truncatula* (**a**) and barley (**b**) genes upregulated by either N and/or P starvation, repressed in *nsp1* or *nsp2* mutants in at least **b** one nutrient condition and activated by overexpression of *miRR-MtNSP2* in *M. truncatula* (**a**) and *MtNSP2* in barley (**b**). +P-N, -P+N and -P-N represent the expression of these starvation-induced genes in wild-type plants by comparing -N or/and -P conditions to +P+N conditions. *nsp* show gene expression in *nsp* mutants compared to wild-type plants grown in the same nutrient conditions (-N or/and -P), while *NSPox* show *NSP* overexpression roots compared to wild type under nutrient replete conditions. Genes are clustered by expression pattern. A 1.5-fold cutoff was used for log2 fold changes with all the heatmaps.

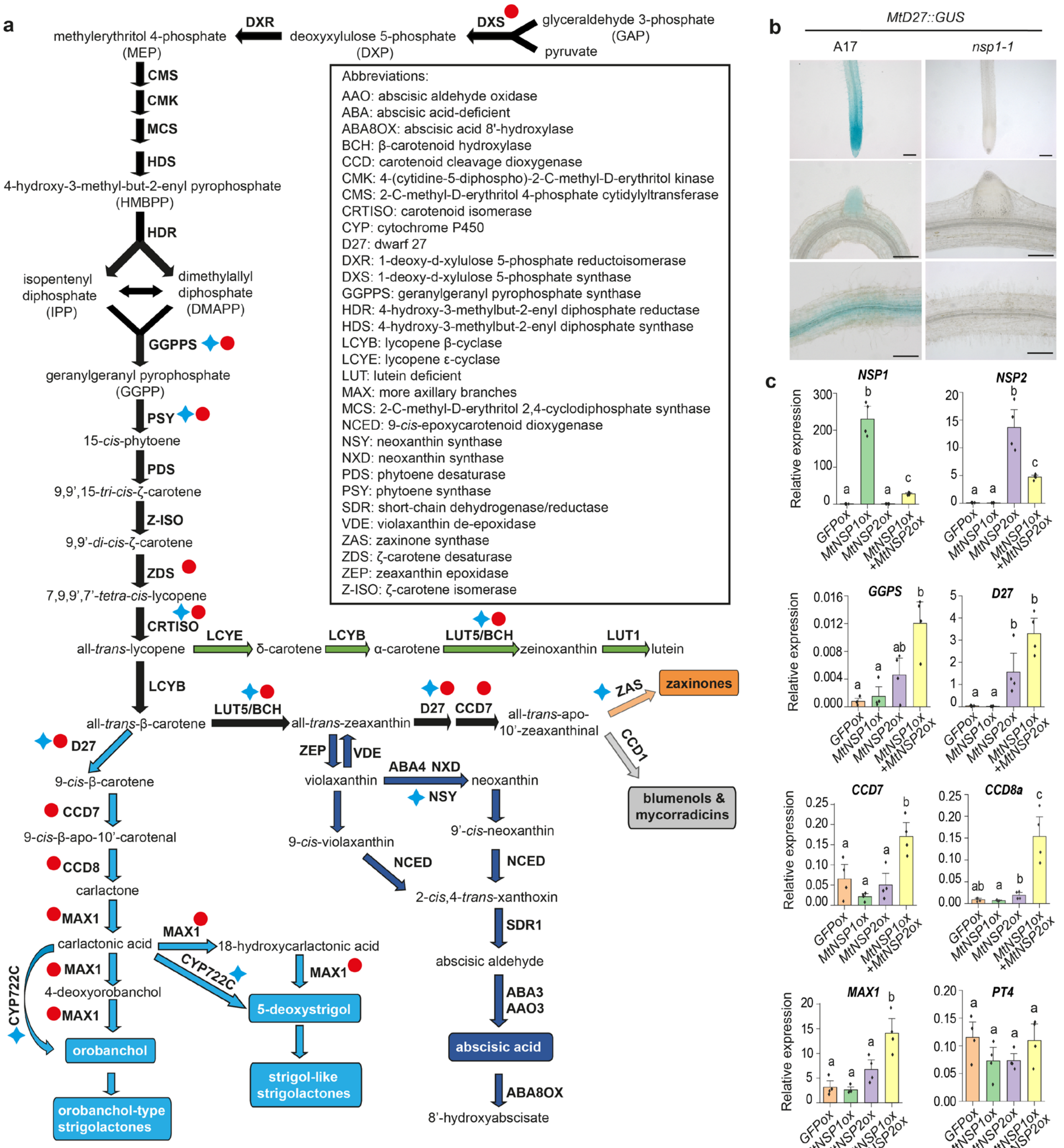

Supplementary Figure 8. *NSP1* and *NSP2*-regulated genes involve the apocarotenoid biosynthetic pathway. a A subset of the biosynthetic pathway of apocarotenoids in plants. The enzymes in each step are highlighted in bold and their full names are listed in the inset. The genes regulated by nutrient starvation in an NSP-dependent manner (Fig. 5a, b) are highlighted on the pathway, with blue stars representing *M. truncatula* genes and red circles representing barley genes. **b** Root expression *pMtD27::GUS* in *M. truncatula* wild type and *nsp1-1* mutants. The plants were grown on modFP plates for 4 weeks after hairy-root transformation. Bars = 200μm. The assay was repeated three times with similar results. **c** Expression levels of strigolactone biosynthetic genes in *M. truncatula* roots with overexpression of *GFP*, *NSP1*, *NSP2* and a combination of *NSP1* and *NSP2*, using *PT4* as a negative control. The plants were grown on modFP plates for 4 weeks after hairy-root transformation of the respective construct. Bars represent means of 4 biological replicates. ± s.e.m. Different letters indicate different statistical groups (ANOVA, post hoc Tukey, P < 0.05).

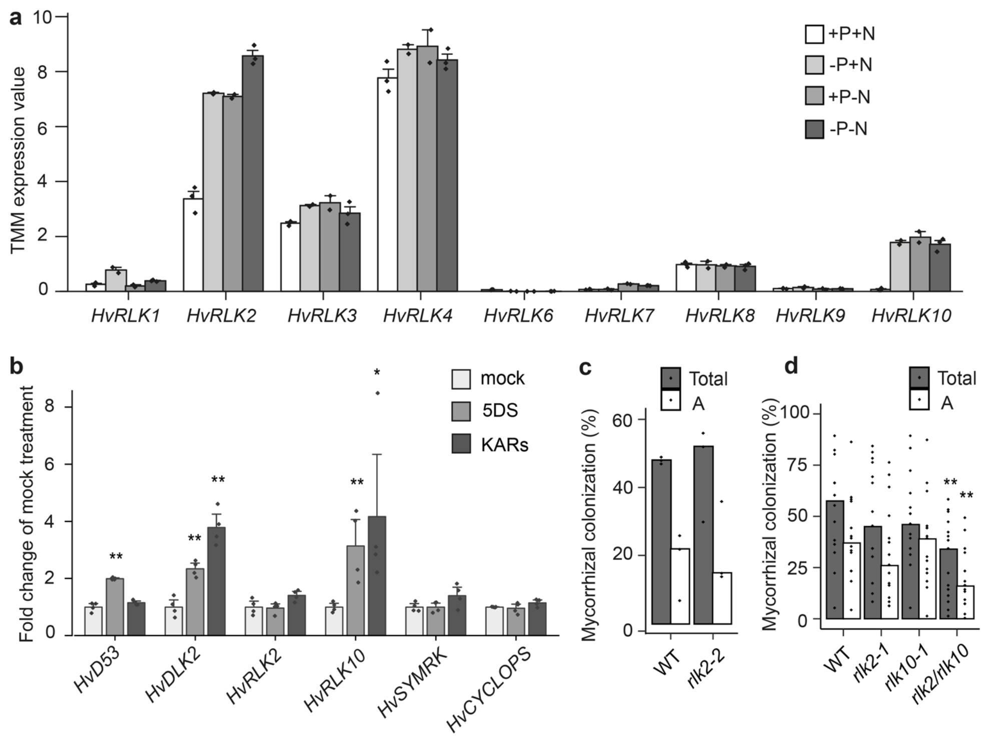

Supplementary Figure 9. Nutrient regulation of barley *RLK* genes via 5DS and KARs. a Relative expression of barley *RLK* genes in wild-type roots grown under different nutrient conditions. The expression values were obtained by conducting TMM normalization from transcriptome data[91]. n=2-3 biologically independent samples. ±s.e.m. **b** qPCR showing regulation of symbiosis genes by 5DS and KARs. Wild type plants were grown under repressive P-conditions and pretreated for 2 days on solid media containing 1 μM 5-deoxystrigol (5DS) or a mixture of 1 μM karrikin 1 and karrikin 2 (KARs). *D53* acts as a marker gene for strigolactone treatment[112], while *DLK2* responding to both strigolactone and karrikin treatment[113]. n=4 biologically independent samples, ±s.e.m.; **indicates $p<0.01$, *indicates $0.01<p<0.05$, measured using a Student's *t*-test (one-tailed, two-sample equal variance). **c** Root-length colonization of barley wild type and *rlk2-2* mutant grown under low P conditions. The colonization levels were measured at 5 weeks post inoculation. n=3 biologically independent samples. **d**. A repeat of Fig. 6e, showing root-length colonization of barley *rlk2*, *rlk10* and *rlk2/rlk10* double mutants grown under P-limited conditions, measured at 7 weeks post inoculation. The *RLK10* mutation in the *rlk2/rlk10* double mutant is equivalent to *rlk10-1*. n=14-15 biologically independent samples. Total: total colonization; A: arbuscules. *p*-values for colonization levels were determined by a one-sided Wilcoxon test. **: $p<0.01$.

Supplementary Table 1. Time course analysis of barley root cells responding to 10^{-7} M *Sm*LCO after incubation with 5DS and KARs.

Incubation time (hr)	10^{-7} M 5DS	10^{-7} M KARs
1	0 / 21	0 / 29
2	0 / 31	0 / 27
3	0 / 28	0 / 26
4	0 / 29	0 / 27
8	0 / 30	3 / 25
16	25 / 37	22 / 31

Cells spiking / total cells

Supplementary Table 2. *NSP1* and *NSP2* percentage dependencies of nutrient upregulated DEGs in *M. truncatula* and barley

Medicago

Condition	-N+P 15D	+N-P 15D	-N-P 15D
**nutrient upregulated and dependent on Mt*NSP1***	332	173	181
**nutrient upregulated and dependent on Mt*NSP2***	442	366	395
**nutrient upregulated**	3635	1041	4047
***MtNSP1*-dependent genes of those nutrient upregulated (%)**	9.1	16.6	4.5
***MtNSP2*-dependent genes of those nutrient upregulated (%)**	12.2	35.2	9.8
**nutrient upregulated and dependent on *MtNSP1* in at least one starvation condition**	588		
**nutrient upregulated and dependent on *MtNSP2* in at least one starvation condition**	900		
**nutrient upregulated in at least one starvation condition**	5110		
***MtNSP1*-dependent genes of those nutrient upregulated in at least one starvation condition (%)**	11.5		
***MtNSP2*-dependent genes of those nutrient upregulated in at least one starvation condition (%)**	17.6		

Barley

Condition	-N+P 21D	+N-P 21D	-N-P 21D
**nutrient upregulated and dependent on *HvNSP1***	440	536	999
**nutrient upregulated and dependent on *HvNSP2***	437	1046	1404
**nutrient upregulated**	5932	5648	7009
***HvNSP1*-dependent genes of those nutrient upregulated (%)**	7.4	9.5	14.3
***HvNSP2*-dependent genes of those nutrient upregulated (%)**	7.4	18.5	20.0
**nutrient upregulated and dependent on *HvNSP1* in at least one starvation condition**	1576		
**nutrient upregulated and dependent on *HvNSP2* in at least one starvation condition**	2436		
**nutrient upregulated in at least one starvation condition**	10412		
***HvNSP1*-dependent genes of those nutrient upregulated in at least one starvation condition (%)**	15.1		
***HvNSP2*-dependent genes of those nutrient upregulated in at least one starvation condition (%)**	23.4		

Supplementary Table 3. List of constructs

L1 plasmids for barley transformation

ENSA ID	ENSA Standard name	Backbone	PU	S	C	SC	T
EC15567	pL1M-R5-pZmUBI-MtNSP1-FLAG-tAg7-15567	EC47841 pL1V-R5	EC15455 pZmUBI-intron	EC15196 MtNSP1	EC15198 FLAG	-	EC15319 tAg7
EC15568	pL1M-R5-pOsUBI3-MtNSP2-FLAG-tRbcS-15568	EC47841 pL1V-R5	EC15328 pOsUBI3-intron	EC15197 MtNSP2	EC15198 FLAG	-	EC15318 tRbcS
EC15031	pL1M-R2-pNFBx4-NLS-cyPET-t35S-15031	EC47811 pL1V-R2	EC15059 pNFBx4	EC15101 NLS	EC15096 cyPET	-	EC41414 t35S
EC15034	pL1M-R3-pAtUBI10-dsRed-tNOS-15034	EC47822 pL1V-R3	EC15062 pAtUBI10	-	-	EC15073 dsRed	EC41421 tNOS
EC15571	pL1M-R4-pINF-NLS-yPET-tActin2-15571	EC47831 pL1V-R4	EC15249 pINF	EC15101 NLS	EC15098 yPET	-	EC44300 tActin2

L2 plasmids for barley transformation

ENSA ID	ENSA Standard name	2i-1 Backbone	position 1	position 2	position 3	position 4	position 5	End linker
EC15027	pL2V-HYG-15027	EC50505 pL2V-1	EC15030 p35S-HYG-tNOS	-	-	-	-	EC49255 pELB-1
EC15551	pL2B-HYG-MtNSP1-FLAG-cyPyP-15551	EC15027 pL2V-HYG		EC15031 pNFBx4-NLS-cyPET	EC15034 pAtUBI-dsRed	EC15571 pINF-NLS-yPET	EC15567 pZmUBI-MtNSP1-FLAG	EC41800 pELE-5
EC15552	pL2B-HYG-MtNSP2-FLAG-cyPyP-15552	EC15027 pL2V-HYG		EC15031 pNFBx4-NLS-cyPET	EC15034 pAtUBI-dsRed	EC15571 pINF-NLS-yPET	EC15568 pOsUBI3-MtNSP2-FLAG	EC41800 pELE-5

L1 plasmids for *Medicago* hairy-root transformation

ENSA ID	ENSA Standard name	Backbone	PU	S	C	T
EC22522	pL1M-R2-pLjUBI1-3xFLAG-MtNSP1-tOCS-22522	EC47811 pL1V-R2	EC15251 pLjUBI1	EC58028-pL0M-S-3XFlag	EC15208 pL0M-C-MtNSP1	EC41432 tocs
EC22523	pL1M-R2-pBdEF1a-3xMyc-MtNSP2-t35S-22523	EC47811 pL1V-R2	EC15336 pBdEF1a	EC15213-pL0M-S-3xMyc	EC15210 pL0M-C-MtNSP2	EC41414 t35S
EC43038	pL1M-R2-pBdEF1a-3xMyc-MtNSP2-miRR-t35S-43038	EC47811 pL1V-R2	EC15336 pBdEF1a	EC15213-pL0M-S-3xMyc	EC43020-pL0M-C-MtNSP2-miRR	EC41414 t35S
EC59216	pL1M-R1-pAtUBI10-RUBY-tNOS-59216	EC47802 pL1V-R1	EC15062 pAtUBI10	EC59214-pL0M-SC3-RUBY-N	EC59215-pL0M-C 4-RUBY-C	EC41421 tNos

L2 plasmids for *Medicago* hairy-root transformation

ENSA ID	ENSA Standard name	2i-1 Backbone	position 1	position 2	End linker
EC59234	pL2B-RUBY-EV-59234	EC50507-L2	EC59216-R1-pAtUBI10-RUBY-tNOS	-	EC41722 pELE-1
EC59217	pL2B-RUBY-pLjUBI-3xFLAG-MtNSP1-59217	EC50507-L2	EC59216-R1-pAtUBI10-RUBY-tNOS	EC22522-R2-pLjUBI1-3xFLAG-MtNSP1-tOCS	EC41744 pELE-2
EC59218	pL2B-RUBY-pBdEF1a-3xMyc-MtNSP2-59218	EC50507-L2	EC59216-R1-pAtUBI10-RUBY-tNOS	EC22523-R2-pBdEF1a-3xMyc-MtNSP2-t35S	EC41744 pELE-2
EC59220	pL2B-RUBY-pBdEF1a-3xMyc-MtNSP2-miRR-59220	EC50507-L2	EC59216-R1-pAtUBI10-RUBY-tNOS	EC43038-R2-pBdEF1a-3xMyc-MtNSP2-miRR-t35S	EC41744 pELE-2

Supplementary Table 4. Primer sequences

Primers for quantitative PCR

MtHistone-F	ATTCCAAAGGCGGCTGCATA
MtHistone-R	CTTTGCTTGGTGCTGTTTAGATGG
MtNSP1-qPCR-F	GCGATTTCGCCACTGGATTC
MtNSP1-qPCR-R	CAGCCTCGCCTTCCATCATT
MtNSP2-qPCR-F	GTCCTCGAACAGCTCAGTCC
MtNSP2-qPCR-R	GCGTTTTTATTGCCGTTGTT
MtUbiquitin-F	GCAGATAGACACGCTGGGA
MtUbiquitin-R	AACTCTTGGGCAGGCAATAA
MtD27-F	GAGATGATATTCGGCCAGGAAC
MtD27-R	GCATGGTTTTTCTTAGCCTTGC
MtCCD7-F	GATGTGGGGGAAGAAGCTATTG
MtCCD7-R	TCCCAATCGTATCCAACGTG
MtCCD8-F	GAAGATGGGAGGGTAACTGCTG
MtCCD8-R	AGAACATCTTCGCCGTTAAATG
MtGGPS-F	TGTCCGTCCGGTACTCTGTATTGC
MtGGPS-R	CGCATGCCGATGGAATTGATGC
MtMAX1-F	AGGTTCCTGGTCCACCATCT
MtMAX1-R	GCTGTCTTCCCATATGAAATCTGT
MtPT4-F	GACACGAGGCGCTTTCATAGCAGC
MtPT4-R	GTCATCGCAGCTGGAACAGCACCG
HvADP-F	GCTCTCCAACAACATTGCCAAC
HvADP-R	GAGACATCCAGCATCATTCATTCC
HvD53-F	AACGGCGATCTGTCATCTTC
HvD53-R	AGTCTCGGAATCTACCAGTCTTA
HvDLK2-F	GTACATTAACTCGGAGGAGGAG
HvDLK2-R	CTTGACCCAGGCTTGGAA
HvRLK2-F	CAACCGCACCCACACTT
HvRLK2-R	GCAGCTCGGAGGTGAAATAC
HvRLK10-F	GCAGTTCATCGACAAGCCC
HvRLK10-R	GACACGTCGCCTTTCGCTGG
HvSYMRK-F	CTGACGGAGCTAACTGAGATTG
HvSYMRK-R	GGTGAGTGAGAGCAGAGAAAG
HvCYCLOPS-F	AGCAGGTGCCTTTCCTAATC
HvCYCLOPS-R	GTGGTTGCTGGTTATGGTTATTC

Primers for genotyping of *Medicago Tnt1* mutants

Tnt-F1	TCCTTGTTGGATTGGTAGCCAACTTTGTTG
Tnt-R1	TGTAGCACCGAGATACGGTAATTAACAAGA
MtNSP1-NF9220-F1	GTCTCTTTCTTTCCATCATTTTTG
MtNSP1-NF9220-R1	GGCTCTTCCACCATAGTTCC
MtNSP2-NF10950-F	ATGCCATCAATGACCTCCACT
MtNSP2-NF10950-R	TATTACTACCCACACCCACCTCTT
MtNSP2-LIKE-NF17492-F	TCACTCACCAACGACGAAATC
MtNSP2-LIKE-NF17492-R	TCAAGTAACCAACAAGACAACAAT
CCD7-NF1485-F	CGAGGAGTATGACCGAAAGACG
CCD7-NF1485-R	GCCCAACCAAAGCACCTAAAT
CCD8-NF18323&11036-F	TGACGGAGACACAAAAAG
CCD8-NF18323&11036-R	ACATGAATTAATTACTGACAAGAG
D14-NF18262-F	ATCTAGAGTGGTTTAACGAGTGTG
D14-NF18262-R	AGGCTTCATAGTTTGCTTCCATTG
D14La-NF13623-F	GACAGGGACTACTTTGGAGGATTT
D14La-NF13623-R	TATTTCACATGACCAACAAGACAA
D14Lb-NF5873-F	TGATAACATGGGTGCTGGTA
D14Lb-NF5873-R	TGCCGAAATTACCTCACAACTAT
MAX2-NF18520-F	ATACTACCACAGCCGCCTCACTCC
MAX2-NF18520-R	GACGCCTATTCAACGCATCTTCA
SMAX1-NF4373-F	AAGGAATTAGGGGAGGGTGTAT
SMAX1-NF4373-R	TGGCGTAGGTGTCAAGGTCT

Primers for semi-quantitative RT-PCR

MtNSP1-RT-F	GCCACAAATAGCACAACCAACA
MtNSP1-RT-R	CGAAATCGCCACAACTACTGC
MtNSP2-RT-F	ATGCCATCAATGACCTCCACT
MtNSP2-RT-R	TATTACTACCCACACCCACCTCTT
MtNSP2L-RT-F	GGCCCACATCTTCGCATCAC
MtNSP2L-RT-R	TACAAGTCCAAACAGAAGCAGAAA
CCD7-RT-F	CATTCCCAACCCCTCATCTTACA
CCD7-RT-R	CCCCACATACTGCTCCCATTG
CCD8a-RT-F	GCGTGGTGAAGCTCGGTGATG
CCD8a-RT-R	GTTCTTAGCCCTTTCTGATTGTAG
D14-RT-F	CGTAGAAGGCTCCGGCGACAAATA
D14-RT-R	ACTGCACCGTACTCTTCCCACCAA
D14La-RT-F	GACAGGGACTACTTTGGAGGATTT
D14La-RT-R	TATTTCACATGACCAACAAGACAA
D14Lb-RT-F	TTGCATATTTGTTGGTCATTCTGT
D14Lb-RT-R	ATCTGCCTCCATTTTCATCAAG
MAX2-RT-F	ATACTACCACAGCCGCCTCACTCC
MAX2-RT-R	GACGCCTATTCAACGCATCTTCA
SMAX1-RT-F	AAGGAATTAGGGGAGGGTGTAT
SMAX1-RT-R	TGGCGTAGGTGTCAAGGTCT

Supplementary Table 5. Generation of barley CRISPR mutants

Mutants	Guide A	Guide B	Deletion sites
symrk-1	ccccatctgcctgggaaggcttc	gggccaattcccgccgccatcgg	a 2 bp deletion at + 94-95 bp and a 31 bp deletion from +719 bp to +749 bp
ccamk-1	ggtttctccatagtgagaagagg	gcgatgatggggatgcagcaggg	a 4 bp deletion from +166 bp to +169 bp
ccamk-2	gtcacagatgtcctcggccgagg	ggcttggccctgcgatgatgggg	a 1 bp deletion at +58 bp
cyclops-1	ggaggagttcatggagatggagg	gccgccgagcatggagatgatgg	a 1 bp deletion at +8 bp and a 35 bp deletion from +113 bp to +147 bp
cyclops-2	ggaggagttcatggagatggagg	gccgccgagcatggagatgatgg	a 4 bp deletion from +8 bp to +11 bp and a 2bp deletion at +114-115 bp
ram1-1	ggagggacttcagccctctgagg	ggcaaagcctgaccagtgggcgg	a 1 bp insertion at +961 bp
ram1-2	ccaacctctacaacaacagcacc	gtcagtaatacagtcagatcagg	a 1 bp insertion at +1013 bp
nsp1-1	gtcgccgctcgtggcgacga	gacgctcccaatgtcatcct	a 1 bp deletion at +164 bp
nsp1-2	gtccaggccgtcgccgctcg	gcccaggaagcgcaagtctc	a 4 bp deletion from +286 bp to +289 bp
nsp2-1	gtgggggatctcgagatctccgg	gcacgacgacgacctgccgcagg	a 314 bp deletion from +48 bp to +361 bp
nsp2-2	gtgggggatctcgagatctccgg	gcacgacgacgacctgccgcagg	a 3 bp deletion from +38 bp to +40 bp and a 1 bp insertion at +356 bp
rlk2-1	gcagaacctcacgcagtaccagg	gtgccaaccaagctcgaggtcgg	a 1 bp insertion at +678 bp
rlk2-2	gcagaacctcacgcagtaccagg	gtgccaaccaagctcgaggtcgg	a 1 bp insertion at +628 bp and a 1 bp insertion at +678 bp
rlk10-1	cccgccgcttcctctgctgctgc	ggacctcttcggcgtgagccggg	a 1 bp insertion at +14 bp
rlk10-2	cccgccgcttcctctgctgctgc	ggacctcttcggcgtgagccggg	a 1 bp insertion at +273 bp
d14l-1	gacatagccctgagcgttgc	tcgttgaggtgatgccctcc	a 32 bp deletion from +88 bp to +119 bp
d14l-2	aactctcttcaacattcgcc	tcctaccactcgtctcggtc	a 1 bp insertion at +169 bp
